# Weakening synergies in carbon and air pollution co-control necessitate robust structural transitions in China’s transportation sector

**DOI:** 10.1093/nsr/nwaf422

**Published:** 2025-09-27

**Authors:** Zhulin Qi, Yixuan Zheng, Wenxin Cao, Yuxi Liu, Xin Li, Yueying Fei, Shigan Liu, Fangming Jiang, Chuchu Chen, Yueyi Feng, Zhang Wen, Xuying Wang, Yu Lei, Zhibin Wang, Gang Yan, Jinnan Wang

**Affiliations:** College of Environmental & Resource Sciences, Zhejiang University, Hangzhou 310058, China; Key Laboratory of Environmental Pollution and Greenhouse Gases Co-control, Ministry of Ecology and Environment, Chinese Academy of Environmental Planning, Beijing 100041, China; Key Laboratory of Environmental Pollution and Greenhouse Gases Co-control, Ministry of Ecology and Environment, Chinese Academy of Environmental Planning, Beijing 100041, China; Key Laboratory of Environmental Pollution and Greenhouse Gases Co-control, Ministry of Ecology and Environment, Chinese Academy of Environmental Planning, Beijing 100041, China; College of New Energy and Environment, Jilin University, Changchun 130012, China; Key Laboratory of Environmental Pollution and Greenhouse Gases Co-control, Ministry of Ecology and Environment, Chinese Academy of Environmental Planning, Beijing 100041, China; Department of Environmental Science and Engineering, Beijing Technology and Business University, Beijing 100048, China; Department of Environmental Science and Engineering, Beijing Technology and Business University, Beijing 100048, China; Department of Earth System Science, Ministry of Education Key Laboratory for Earth System Modeling, Institute for Global Change Studies, Tsinghua University, Beijing 100084, China; College of Environmental & Resource Sciences, Zhejiang University, Hangzhou 310058, China; Key Laboratory of Environmental Pollution and Greenhouse Gases Co-control, Ministry of Ecology and Environment, Chinese Academy of Environmental Planning, Beijing 100041, China; Key Laboratory of Environmental Pollution and Greenhouse Gases Co-control, Ministry of Ecology and Environment, Chinese Academy of Environmental Planning, Beijing 100041, China; Key Laboratory of Environmental Pollution and Greenhouse Gases Co-control, Ministry of Ecology and Environment, Chinese Academy of Environmental Planning, Beijing 100041, China; Key Laboratory of Environmental Pollution and Greenhouse Gases Co-control, Ministry of Ecology and Environment, Chinese Academy of Environmental Planning, Beijing 100041, China; Key Laboratory of Environmental Pollution and Greenhouse Gases Co-control, Ministry of Ecology and Environment, Chinese Academy of Environmental Planning, Beijing 100041, China; Key Laboratory of Environmental Pollution and Greenhouse Gases Co-control, Ministry of Ecology and Environment, Chinese Academy of Environmental Planning, Beijing 100041, China; College of Environmental & Resource Sciences, Zhejiang University, Hangzhou 310058, China; Key Laboratory of Environmental Pollution and Greenhouse Gases Co-control, Ministry of Ecology and Environment, Chinese Academy of Environmental Planning, Beijing 100041, China; Key Laboratory of Environmental Pollution and Greenhouse Gases Co-control, Ministry of Ecology and Environment, Chinese Academy of Environmental Planning, Beijing 100041, China; College of Environmental & Resource Sciences, Zhejiang University, Hangzhou 310058, China

**Keywords:** co-control strategy, GHG emissions, clean air, public health, on-road transportation

## Abstract

A fossil fuel-dominated country like China is promoting a policy shift toward synergistic governance integrating climate change mitigation and air quality improvement, especially in the on-road transportation sector. However, the efficacy of current emission control policies in mitigating greenhouse gas (GHG) emissions and air pollution remains poorly quantified through a synergy-focused lens. Here, we develop an integrated analytical framework incorporating a Synergy Index to evaluate policy performance across GHG abatement and air pollution-related health burden reduction. We find that China’s on-road environmental policies reduced GHG emissions by 427 Mt CO_2_e and averted 104 000 premature mortalities during 2010–2015, but these gains declined to 278 Mt CO_2_e and 72 000 mortalities in 2015–2020, marking an 18.7% decline in the overall Synergy Index. Policy-specific evaluations reveal that traditional policies like tightening emission standards and fuel quality and removing high-emitting vehicles drove early synergies. Conversely, emerging structural transitions, including promoting electric vehicles and a modal shift from road to more efficient modes, grew in prominence but failed to offset declining efficacy. Strategic optimization of vehicle fleet and transportation structure to meet 2025 targets could reverse this declining trend. These findings underscore the urgency of accelerating structural transitions to sustain effective carbon and pollution co-control in the transportation sector, with global relevance for fossil fuel-dependent economies.

## Teaser

China's transportation emission control policies have yielded substantial carbon mitigation, clean air, and public health benefits during 2010–2020, but their synergies are weakening, making robust structural transitions essential for sustained carbon and air-pollution co-control.

## INTRODUCTION

As societies remain entrenched in fossil fuel-driven development, interconnected sustainability challenges, such as global warming, air pollution, and associated health damages, pose formidable barriers to achieving global Sustainable Development Goals (SDGs) [1,2]. The shared sources of greenhouse gas (GHG) and air pollution emissions provide a unique opportunity to address issues through integrated solutions and enhance environmental and public health welfare [3,4]. Accordingly, the State Council of China has enacted the ‘Carbon Peaking and Carbon Neutrality’ and ‘Beautiful China’ goals aimed at accelerating its environmental governance targeting carbon-pollution co-control and broader sustainable objectives [5].

On-road transportation stands as a critical sector for carbon-pollution co-control. The sector is responsible for nearly 16% of global CO_2_ emissions and about 19% of anthropogenic BC emissions [6–8], with BC being a key short-lived climate pollutant (SLCP). As the dominant contributor to transport-related SLCP CO_2_e (∼90%) [9,10], BC plays a central role in shaping the sector’s near-term climate impacts. The sector also accounts for 5%–7% of the global disease burden associated with ambient particulate matter (PM_2.5_) and ozone (O_3_) pollution [11–13]. Mitigation of on-road transportation emissions therefore provides an effective pathway to deliver substantial near-term climate and health benefits while securing long-term CO_2_ reductions.

**Figure 1.**
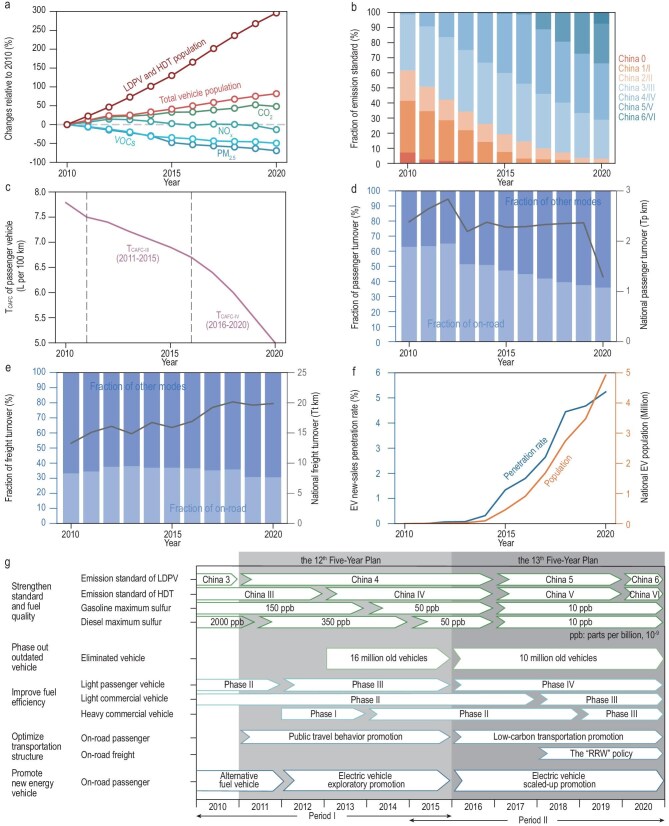
Trends in key socioeconomic and policy factors affecting emissions from China’s on-road transportation sector from 2010 to 2020. (a) Relative changes in vehicle population, as well as CO_2_ and major air pollutant emissions from on-road vehicles. (b) Fraction of vehicles complying with specified emission standards. (c) Trends in national corporate average fuel consumption (CAFC) targets for light-duty passenger vehicles. (d) National passenger turnover and fraction of different modes. (e) National freight turnover and fraction of different modes. (f) National electric vehicle population and new-sales penetration rate. (g) Progress of major environmental policies for China’s on-road transportation sector. Detailed information regarding each policy is documented in the Supplementary Information.

China is the world’s largest vehicle market and a key proponent of carbon-pollution co-control [14–16], with its on-road vehicle fleet expanding from 178.9 million to 325.4 million between 2010 and 2020, dominated by light-duty passenger vehicles and heavy-duty trucks that emit most CO_2_ and co-emitted air pollutants (Fig. 1a). In response, China has enacted a suite of emission control policies to address climate and environmental threats in the on-road transportation sector in recent decades (Fig. 1g). From 2010 to 2020, China strengthened emission controls through *Strengthen Standard and Fuel Quality*, which upgraded emission standards from China 3 to 6 for light-duty passenger vehicles and from China III to VI for heavy-duty trucks, and aligned fuel quality with these requirements. This effort was reinforced by *Phase Out Outdated Vehicle*, removing more than 26 million high-emitting vehicles [17,18] and increasing the share of the fleet meeting China Four or stricter to over 70% by 2020 (Fig. 1b) [19]. To improve energy efficiency, *Improve Fuel Efficiency* was implemented to tighten fuel-economy requirements (Fig. 1c) [20]. At the same time, *Optimize Transportation Structure* shifted mobility patterns, lowering road passenger turnover from 63% to 35.9% and road freight from 33.2% to 30.6% (Fig. 1d, e) [21], while the *Promote New Energy Vehicle* drove exponential growth in clean energy vehicle populations (Fig. 1f) [22]. Further details on these policies are provided in the Supplementary Information, Note S1.

Policy effectiveness evaluation is essential for informing mitigation strategies and enabling timely adjustments to optimize outcomes. Previous studies provide a foundation for estimating the benefits of on-road transportation sector policies, including improvements in air quality and public health from stringent emission standards assessed with atmospheric and epidemiological models [23,24], CO_2_ reductions from fuel efficiency measures within energy carbon frameworks [25,26], and the health co-benefits identified in prospective decarbonization analyses [27–29], though these impacts were evaluated separately. However, clean air actions and carbon mitigation are deeply interconnected, generating multi-dimensional synergies [3,15,30,31]. To date, there has been no unified framework that consistently quantifies both health and climate dimensions to capture the full spectrum of synergies and trade-offs in carbon-air pollution co-control policy design. This gap may leave the realized extent of synergistic benefits, defined as integrated gains in GHG reductions, cleaner air, and improved public health, uncharacterized, thereby risking misalignments between policy actions and national co-control goals. Moreover, as the world’s fastest-growing vehicle market, China has made rapid progress in on-road environmental policies over the past decade, moving from single-objective approaches before 2022 to an integrated framework that explicitly prioritizes both climate and health co-control [18,23,32]; yet, the synergetic characteristics of these policies across different implementation phases have not been systematically evaluated.

Our study aims to evaluate emission control policies in China’s on-road transportation sector from a synergetic perspective and to identify those within the existing control framework that delivered meaningful health and climate benefits, providing guidance for future carbon-air pollution co-control. Here, we develop an analytical framework to evaluate the synergetic impacts of five major emission control policies (Fig. 1g) over two periods (Period Ⅰ: 2010–2015; Period Ⅱ: 2015–2020). This framework integrates a detailed emission inventory [19], a chemical transport model with the source apportionment module (Comprehensive Air Quality Model with Extensions (CAMx-PSAT/OSAT)) [33], an epidemiological concentration-response (C-R) model [34], and a customized Synergy Index (SynI) framework. The Synergy Index is developed into a two-dimensional framework to evaluate carbon-air pollution co-control policies in an integrated way based on two metrics: (1) the climate metric represented by the sum of CO_2_ and BC emissions, and (2) the public health metric represented by avoided premature deaths attributed to PM_2.5_ and O_3_ exposure. Policies achieving greater and balanced benefits across the two metrics are deemed to exhibit higher synergy. With this framework, we provide a consistent assessment of policy-specific synergies between carbon mitigation, air quality improvement, and public health protection. The methodology framework is presented in Fig. S3 and the details of the analytical approach are provided in the Methods and Supplementary Information.

## RESULTS

### Impacts on air quality, health impacts, and GHG emissions

**Figure 2.**
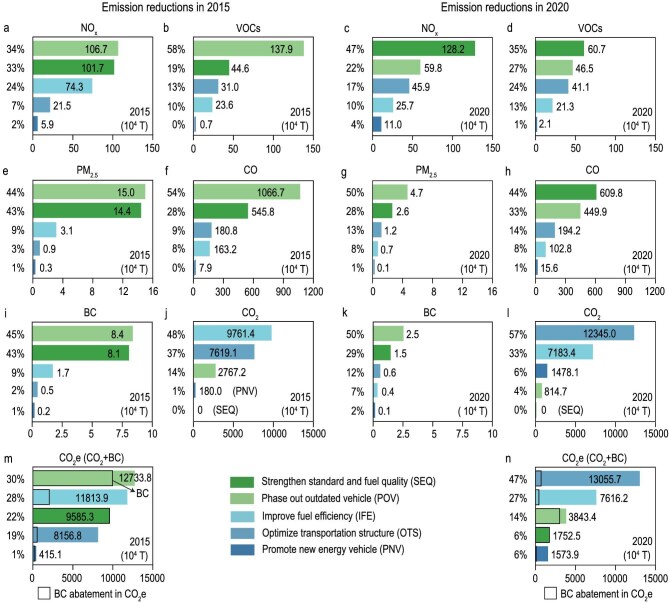
Policy-specific contributions to air pollution and GHG emission reductions. (a–h) Contribution of each policy to reductions in air pollution emissions, including NO*_x_* (a, c), VOCs (b, d), PM_2.5_ (e, g), and CO (f, h), respectively, in 2015 and 2020. (i–n) Contribution of each policy to reductions in GHG emissions, including BC (i, k), CO_2_ (j, l), and total CO_2_e (m, n), respectively, in 2015 and 2020. Numbers on the right of each bar indicate the policy-specific emission reductions, while those on the left represent the fractional contributions to total abatements. Bars outlined in black in (m) and (n) represent CO_2_e emission reductions attributable to BC abatements. The detailed GHG emission reductions are provided in Table S3.

**Figure 3.**
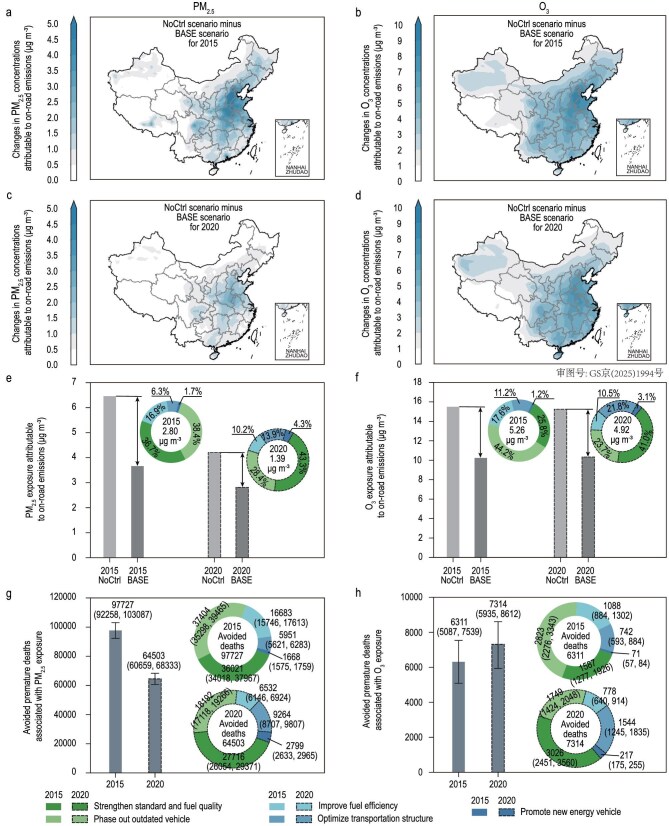
Changes in air quality and associated health impacts attributable to on-road transportation emissions over China in 2015 and 2020 (data for Hong Kong, Macao and Taiwan are not included here). (a, c) Reductions in annual mean PM_2.5_ concentrations attributable to on-road emissions, comparing actual conditions under the current policy status (i.e. BASE scenario in Table S5) with a scenario without emission control policies (i.e. NoCtrl scenario in Table S5) for 2015 (a) and 2020 (c). (b, d) Reductions in average summer MDA8 O_3_ concentrations attributable to on-road emissions, comparing between actual conditions under the current policy status (i.e. BASE scenario in Table S5) and a scenario without emission control policies (i.e. NoCtrl scenario in Table S5) for 2015 (b) and 2020 (d). (e–f) Contribution of each policy to national population-weighted annual mean PM_2.5_ concentrations (e) and population-weighted summer MDA8 O_3_ concentrations (f). (g–h) Policy-specific contributions to avoided premature deaths attributable to PM_2.5_ exposure (g) and O_3_ exposure (h). Dashed-bordered bars and circles in (e–h) represent estimates for 2020. Values in parentheses in (g) and (h) indicate the estimated 95% confidence intervals. Detailed results on air quality improvements and avoided premature deaths are presented in Table S3.

The combined effects of the socioeconomic and policy factors have shaped the trends in air pollution and GHG emissions from China’s on-road transportation sector (Figs 1a and 2). Despite dynamic increases in transportation demand that would ordinarily elevate pollutant emissions, the implementation of five environmental policies from 2010 to 2020 effectively offset these increases (Fig. 1a). For example, these policies are estimated to reduce NO*_x_* emissions by 3.10 Mt (33.9%) in 2015 and by 2.71 Mt (34.6%) in 2020, relative to a non-control scenario (Fig. 2). As a result, the national population-weighted annual mean PM_2.5_ (PWPM_2.5_) and summer maximum daily 8-hour mean O_3_ (PW MDA8 O_3_) concentrations that could be attributable to China’s on-road transportation (herein referred to as attributable air pollution) were estimated to decrease by 2.80 μg/m³ and 5.26 μg/m³, respectively, in 2015, and by 1.39 μg/m³ and 4.92 μg/m³ in 2020 (Fig. 3e–f), during the two periods, respectively, with the most pronounced improvements being observed in eastern China (Fig. 3a–d). Consequently, in 2015, the policies averted 104 038 (95% CI: 97 345–110 626) annual premature deaths related to PM_2.5_ and O_3_, relative to a scenario without five emission control policies; in 2020, the reductions were 71 817 (95% CI: 66 594–76 945) (Fig. 3g–h). The downward trend is primarily attributable to the reduced strength and effectiveness of later policy actions (Fig. 1g), which slowed improvements in PM_2.5_ concentrations and the associated health benefits in terms of avoided premature deaths (Fig. 3e, g).

While CO_2_ emissions continue to rise, the observed decoupling of emission growth from the immense increase in vehicle population suggests progress in emission abatement (Fig. 1a). Overall, the combined impacts of all policies are estimated to have reduced CO_2_ emissions by 203.28 Mt in 2015 and 218.21 Mt in 2020. With BC considered, the total CO_2_-equivalent (CO_2_e) reductions would increase to 427.05 Mt and 278.42 Mt, respectively (Fig. 2i–n), compared to the scenarios without policy interventions, emphasizing the additional GHG reductions from BC abatements. These synergetic climate, environmental, and public health benefits underscore the potential for carbon and air pollution co-control in China’s on-road transportation sector.

Figures 2 and 3 further detail the policy-specific impacts. Among the five control policies, the *Strengthen Standard and Fuel Quality* policy was formulated with the primary goal of reducing air pollutants, whereas the other four policies contributed to both CO_2_ and air pollutant abatement (Fig. 2). The effectiveness of each policy is discussed below.

**Strengthen Standard and Fuel Quality.** This policy played a pivotal role in improving air quality and reducing premature deaths. In 2015, it prevented 37 608 premature deaths (95% CI: 35 295–39 893, 36.1% of total avoided deaths) by reducing PWPM_2.5_ by 1.03 μg/m³ (36.7% of total improvement) and PW MDA8 O_3_ by 1.35 μg/m³ (25.8% of total improvement) (Fig. 3e–h, dark green bar). In the subsequent period, however, while end-of-pipe treatments became increasingly stringent in improving the emission efficiency of NO*_x_* and VOCs, the intensity of efforts to further reduce primary PM_2.5_ emissions, which are a major PM_2.5_ precursor, weakened (Fig. 2, dark green bar) [18,35]. As a result, the PWPM_2.5_ improvement slowed, leading to a reduction in the total number of avoided deaths (Fig. 3e, g). It should be noted that, although the *Strengthen Standard and Fuel Quality* was primarily designed for air pollutant control, it substantially reduced BC emissions by 43% and 29% in 2015 and 2020, respectively (Fig. 2i, k). Expressed in CO_2_-equivalents, these BC reductions make a meaningful contribution to climate mitigation and highlight the importance of addressing short-lived climate pollutants in control policies.

**Phase Out Outdated Vehicle.** This action was a notable contributor to air quality improvement and GHG reductions. In Period I, with 16 million high-emitting vehicles eliminated (Fig. 1g), the policy contributed to 40 227 avoided deaths (95% CI: 37 574–42 808, 38.7%) as a result of a 1.07 μg/m³ reduction in annual PWPM_2.5_ and a 2.33 μg/m³ reduction in summer PW MDA8 O_3_ in 2015 (Fig. 3e–h, light green bar). It was also the largest contributor to CO_2_e emission reductions, accounting for 29.8% of total abatements (Fig. 2m, light green bar). However, as the fleet became cleaner with the retirement of the highest-emitting vehicles, subsequent actions targeted those that met stricter standards, limiting the remaining mitigation potential. In Period II, both health benefits and CO_2_e abatement decreased due to a 70% reduction in the number of vehicles eliminated (Fig. 1g). In particular, CO_2_e abatement in the second period was ∼30% of that in the first one (Fig. 2m–n).

**Improve Fuel Efficiency.** This measure played a key role in CO_2_e emission abatement by lowering fuel consumption per unit of distance, contributing to ∼27% of total CO_2_e reductions in both periods (Fig. 2m–n, light blue bar). The decreased fuel consumption also curtailed emissions of air pollutants, such as NO*_x_*, VOCs, and primary PM_2.5_, thereby improving air quality and mitigating health impacts (Figs 2 and 3). For example, in 2015, the policy averted 17 771 (95% CI: 16 630–18 915, 17.1%) premature deaths via reductions of 0.47 μg/m^3^ in annual PWPM_2.5_ and 0.93 μg/m^3^ in summer PW MDA8 O_3_ (Fig. 3e–h, light blue bar).

**Optimize Transportation Structure.** The policy, which promotes shifts in transportation modes, demonstrated increasing synergetic benefits over time (Figs 2 and 3, medium blue bar). In Period I, its impact on PM_2.5_ and NO*_x_* emissions was limited (Fig. 2a, e), as structural optimization efforts primarily targeted the passenger transport subsector, leaving heavy-duty trucks (HDTs) in the freight subsector—the primary emitters—largely overlooked. With subsequent structural adjustments in the freight sector, the policy’s effectiveness improved markedly, with avoided premature deaths and CO_2_e abatements increasing by ∼60% relative to the first period. In 2020, the policy was estimated to have averted 10 808 (95% CI: 9952–11 642, 15%) premature deaths and reduced CO_2_e emissions by 130.56 Mt (46.9% of the total reduction) (Fig. 2n and Fig. 3g–h).

**Promote New Energy Vehicle.** Intended to reduce reliance on ICEVs and support carbon-pollution co-control, this policy has achieved limited positive impact during 2010 to 2020 (Figs 2 and 3, dark blue bar). This limited progress can be attributed to early technological constraints, which led to the prioritization of natural gas and hybrid vehicles in public transit (such as buses and taxis) [36]. Although subsequent efforts shifted toward promoting pure EVs [37], substantial private-sector adoption occurred only recently [25], resulting in a modest new-sales EV penetration rate of just 5.2% in 2020 (Fig. 1f).

### Policy-specific synergetic benefits

**Figure 4.**
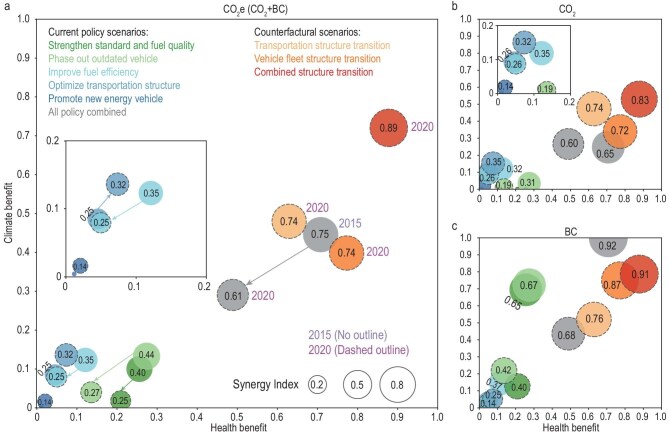
Policy-specific synergy levels in GHG and air pollution emission co-control. (a–c) Results for the case that climate metrics are represented by CO_2_e (a), CO_2_ (b), and BC (c) emission reductions, respectively. The x-axis represents health metric, showing relative changes in deaths associated with on-road emissions attributed to environmental policies, compared to the baseline attributable deaths in 2015. The y-axis represents climate metric, showing relative changes in CO_2_e emissions attributed to environmental policies, compared to the baseline CO_2_e emissions from on-road transportation in 2015. Bubbles with dashed borders represent results for 2020, with numerical labels corresponding to the estimated Synergy Index. Values for health metric, climate metric, and the estimated Synergy Index range from 0 to 1, with higher values indicating superior performance.

Figures 4 presents the synergetic level of GHG emission and air pollution co-control for each policy over two periods. Overall, China’s on-road emission control policies showed reduced synergies, with the combined Synergy Index dropping by 18.7% from 0.75 in Period I to 0.61 in Period II (Fig. 4a, grey bubble). A strong positive correlation between the Synergy Index of policies and their health and climate dimensions was observed, as both air pollutants and CO_2_ emissions in on-road transportation primarily result from fuel combustion and are therefore reduced simultaneously by policies that influence fuel consumption. In addition, the overall performance of the climate metric (denoted by the y-axis in Fig. 4) consistently lags behind the health metric (denoted by the x-axis in Fig. 4) across both periods. This pattern is primarily due to the stronger and more targeted reductions of pollutants affecting population exposure compared with CO_2_. For example, *Strengthen Standard and Fuel Quality* accounted for 36.1% and 42.8% of total avoided premature deaths in 2015 and 2020 (Fig. 3e–h), respectively, while *Improve Fuel Efficiency* contributed less than 30% of total CO_2_e abatements (Fig. 2i–n). Although the climate metric incorporates BC reductions, which accounted for 52.4% of CO_2_e in 2015 and 21.6% in 2020 (Fig. 2i–n and Fig. S2), overall climate benefits remained weaker than health benefits.

This overall reduction in synergy reflects divergent trends among individual policies (Fig. 4a). Generally, traditional policies, including *Phase Out Outdated Vehicle, Strengthen Standard and Fuel Quality*, and *Improve Fuel Efficiency*, showed declining synergetic benefits, while emerging structural transition policies, including *Optimize Transportation Structure* and *Promote New Energy Vehicle* boosted additional synergies. However, the gains from these emerging policies were insufficient to counterbalance the declines observed in traditional policies.

In Period I, traditional policies dominated the synergy rankings. Specifically, the *Phase Out Outdated Vehicle* policy (Fig. 4a, light green bubble) achieved the highest Synergy Index (0.44) by reducing attributable premature deaths by 27% and CO_2_e emissions by 13% relative to the actual 2015 levels caused by on-road transportation emissions. However, as most high-emitting vehicles were already retired and the remaining fleet complied with stricter standards, the residual mitigation potential of this policy decreased substantially in Period II (Fig. 1g). Similarly, the *Strengthen Standard and Fuel Quality* policy, despite being a primary air pollution control measure, yielded notable synergies in Period I, driven largely by substantial BC abatements from tightened emission standards for diesel trucks. While the above two traditional policies achieved relative high synergies by greater health metrics, the *Improve Fuel Efficiency* obtained high synergies with larger gains in climate metrics.

In contrast, the *Optimize Transportation Structure* policy (Fig. 4a, medium blue bubble) showed marked improvement over time, with its Synergy Index rising from 0.25 in Period I to 0.32 in Period II, making it the top synergetic policy in the latter period. This enhancement was driven primarily by stronger climate benefits relative to health gains. Similarly, the *Promote New Energy Vehicle* policy (Fig. 4a, dark blue bubble) initially exhibited limited synergy but improved over time as EV adoption increased (Fig 1f). These findings underscore that, as baseline emissions decline and the policy context evolves, it is necessary to complement traditional control with accelerated structural transformation to sustain and amplify climate-health synergetic benefits, particularly from a climate governance perspective.

Figure 4c illustrates the synergetic benefits where the climate metric is represented by BC reductions, underscoring its prominent role in shaping short-term climate benefits and highlighting how early-stage policy actions achieved especially strong GHG reductions by BC. It is therefore important to account for the contributions of BC reductions when evaluating climate benefits. Excluding BC reductions from the analysis would substantially decrease the estimated synergies, especially for policies where BC mitigation is pivotal (Fig. 4c). For instance, comparing Fig. 4b and c, the *Strengthen Standard and Fuel Quality* policy would lose its synergies between climate and public health entirely, with both its estimated gains in climate metric and Synergy Index dropping to 0 in the absence of BC (Fig. 2i, j). Likewise, the *Phase Out Outdated Vehicle* policy, which yielded substantial benefits from BC mitigation in Period I (Fig. 2m, n), experienced a dramatic decline in its climate metric and Synergy Index once BC reductions were excluded. Overall, the Synergy Index for Period I would drop by 0.1 points—to 0.65—if BC contributions were overlooked, as BC alone accounted for 52% of the total CO_2_e reductions during this period (Fig. 2m). This finding points to the substantial contribution of BC reductions to near-term climate benefits and their potential to reinforce the momentum of broader decarbonization strategies.

### Emerging role of structural transition

**Figure 5.**
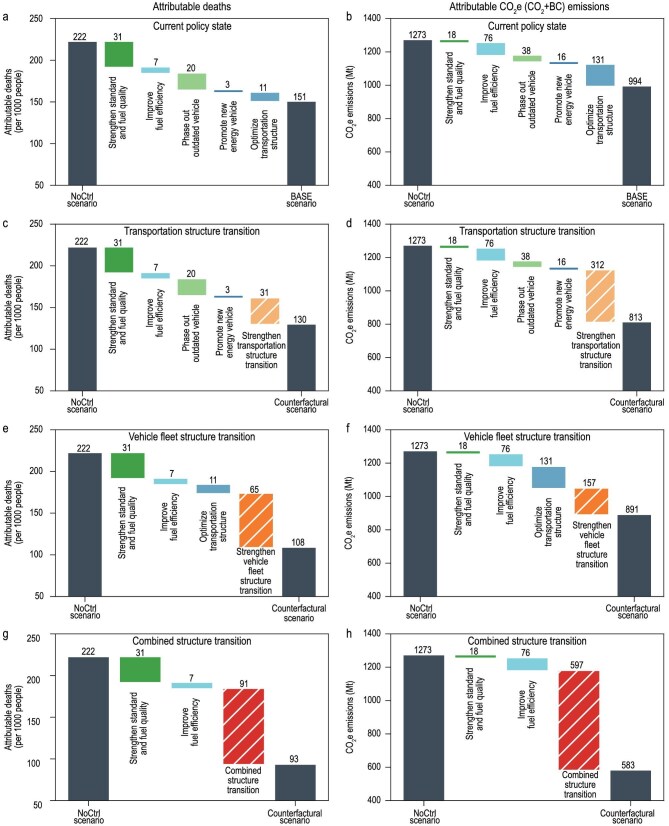
Policy drivers of changes in attributable health impacts and CO_2_e emissions in 2020. (a, c, e, g) Impacts of policies on total premature deaths attributed to PM_2.5_ and O_3_ exposure associated with on-road emissions, in scenarios with the current policy state (a), Strengthen Transportation Structure Transition (c), Strengthen Vehicle Fleet Structure Transition (e), and Combined Structure Transition (g). (b, d, f, h) The impacts of policies on CO_2_e emissions under scenarios with the current policy state (b), Strengthen Transportation Structure Transition (d), Strengthen Vehicle Fleet Structure Transition (f), and Combined Structure Transition (h). The BASE scenarios represent actual conditions under the current policy state; the NoCtrl scenarios indicate conditions in the absence of all emission control policies; the Counterfactual scenarios reflect conditions with enhanced structural transition measures. Uncertainty ranges for the estimated avoided deaths, CO_2_e emission reductions and BC emission reductions (calculated based on CO_2_e) across policy scenarios are presented in Figs S1–S2.

To further explore the potential synergetic gains from emerging structural policies in the near term, we designed three counterfactual scenarios that optimize fleet and transportation structures to meet the 2025 targets, while holding other socioeconomic and policy factors constant at 2020 levels. The results demonstrate that all scenarios substantially enhance overall synergies (Figs 4 and 5). Individually optimizing either the vehicle fleet structure or the transportation structure to the 2025 target (indicated by the orange bubble in Fig. 4a) would increase the overall Synergy Index to 0.74, closely approaching the Period I level of 0.75.

Specifically, the *Strengthen Vehicle Fleet Structure Transition* scenario raises the health metric from 0.49 to 0.77 (increasing avoided deaths from 71 817 to 113 556) and the climate metric from 0.29 to 0.40 (increasing GHG reductions from 278.42 to 381.78 Mt CO_2_e) (Fig. 4a, dark orange bubble and Fig. 5, dark orange bar). In comparison, the *Strengthen Transportation Structure Transition* scenario achieves a different balance of benefits (Fig. 4a, light orange bubble and Fig. 5, light orange bar), with a higher climate metric (0.48, 460.03 Mt CO_2_e emission reduction) but a relatively lower health metric (0.63, 92 472 avoided deaths). This divergence highlights how different structural transitions can be strategically tailored to align with governance priorities, thereby enhancing synergies in diverse ways.

Notably, the *Combined Structure Transition* outperforms all others, achieving a Synergy Index of 0.89—surpassing even the Period I level—with the highest climate metric (0.72, 690.33 Mt CO_2_e emission reduction) and health metric (0.88, 128 993 avoided deaths) across all scenarios (Fig. 4a, red bubble and Fig. 5, red bar). This underscores the transformative potential of simultaneously transitioning both the vehicle fleet and transportation structure, offering a robust pathway for advancing China’s on-road carbon-pollution co-control.

## DISCUSSION AND POLICY IMPLICATIONS

Although substantial progress has been made in fostering synergies between public health protection and climate change mitigation under China’s existing on-road transportation policies, the declining effectiveness of the policies over time underscores the need for systemic changes to pursue multiple goals in the future. The weakening impacts of *Strengthen Standard and Fuel Quality* and *Phase Out Outdated Vehicle* policies during Period II raise concerns about a shrinking emissions baseline and growing implementation challenges as the fleet structure improved. Moreover, the persistent gap between target and actual fuel consumption of ICEVs, especially heavy-duty vehicles [25], has undermined the expected climate benefits of *Improve Fuel Efficiency* and will likely constrain its role in future CO_2_ mitigation. Similar trends are observed in the United States, where the Environmental Protection Agency has reported slowing fuel efficiency gains as driving conditions and consumer demand shifted toward larger vehicles [38]. These concerns are critical for China’s long-term climate goals, as on-road climate mitigation efforts have lagged behind air pollution control by several years [20] and have relied mainly on incremental fuel efficiency improvement. Together, the declining synergies of traditional policies call into question the long-term sustainability of primarily relying on traditional approaches for carbon-pollution co-control.

The observed synergetic gains from the *Optimize Transportation Structure* and *Promote New Energy Vehicle* policies suggest the increasing role of structural strategies as critical complements to traditional approaches in achieving carbon-pollution co-control. Structural strategies are present throughout the policy timeline as part of China’s policy agenda but initially delivered modest benefits because early actions were limited in scale and implementation strength. As national plans have become more specific and ambitious, their scope has broadened and implementation has intensified, with impacts expected to generate even greater synergies. Our study validates the potential of the *Optimize Transportation Structure* policy by demonstrating that reducing on-road passenger and freight turnover below current levels can enhance synergies. Realizing this potential requires prioritizing behavioral changes through expansion of shared mobility services [39–41] and a modal shift in freight transport from roads to railways and waterways through the development of optimized multimodal systems [42,43]. While the *Promote New Energy Vehicle* policy exhibited limited synergies by 2020, we anticipate a notable growth in its benefits as EV adoption accelerates. China’s leadership in the EV market provides a solid basis for the decarbonization of its vehicle fleet and the associated environmental and health co-benefits. The State Council’s target of achieving a 40% EV market share by 2030 was surpassed far ahead of schedule, reaching 47.6% by 2024 [25,44]. As China’s electricity grid continues to transition to cleaner energy sources, the climate and health benefits of electrifying both passenger and truck fleets are projected to grow further [27].

The above structural transformations in the transport sector are increasingly being recognized worldwide for reducing air pollution and CO_2_ emissions [40]. In advanced economies, decades of stringent air pollution controls have delivered substantial improvements in air quality [45,46], allowing structural transitions in the transport sector to be directed primarily toward deep decarbonization. The European Union emphasizes modal shifts toward greener transport and identifies passenger vehicle electrification as a core transition pathway [47,48] while the United States prioritizes multimodal freight hubs and vehicle electrification as foundations for achieving near-zero emissions [49–51]. China, however, faces the dual challenge of meeting national air quality standards while achieving carbon peaking and neutrality targets, making structural transition more complex and more urgent [52]. Our assessment of counterfactual scenarios shows that all structural transition pathways enhance carbon and air-pollution co-control, but their relative advantages differ across health and climate dimensions (Fig. 4a and Fig. S5). The *Strengthen Vehicle Fleet Structure Transition* is better suited to governance priorities that emphasize reducing population exposure and improving public health, whereas the *Strengthen Transportation Structure Transition* is more advantageous when GHG mitigation is the primary objective. The *Combined Structure Transition* provides the strongest overall benefits, underscoring its potential as the most effective long-term strategy. For practical policymaking, this implies that transition pathways could be selected flexibly according to evolving priorities, while progressively moving toward integration to maximize benefits across health and climate domains.

Meanwhile, traditional policies, including *Phase Out Outdated Vehicle, Strengthen Standard and Fuel Quality*, and *Improve Fuel Efficiency*, continue to provide a strong foundation for future co-control efforts despite recent declines in their synergies. Our findings confirm that further implementing the *Phase Out Outdated Vehicle* and *Promote New Energy Vehicle* strategies will yield considerable synergetic benefits by accelerating the transition to a cleaner vehicle fleet. Given that a considerable fraction of emissions is still generated by the existing China 3/III and China 4/IV trucks [19,35], expanding the phase-out list to include all trucks failing to meet China 5/V standards—and promoting their replacement with near-zero-emission vehicles—could accelerate decarbonization and enhance public health benefits. Furthermore, to align newly manufactured ICEVs with national carbon-pollution co-control goals before the comprehensive ban on fuel-powered vehicles, the China 7/VII emission standard is recommended to be introduced at the earliest opportunity during the Fifteenth Five-Year Plan period (15th FYP; 2025–2030) [53]. The European Union has already implemented the Euro 7/VII standard in 2024 to further strengthen the control of conventional air pollutants [54]. Given China’s dual pressures of air pollution control and carbon mitigation, the China 7/VII standard is expected to extend beyond pollutant abatement to also incorporate explicit CO_2_ limits within the vehicle standard framework. Such a dual-track upgrade would integrate air pollutant and carbon constraints into a unified system, reinforcing national efforts to achieve simultaneous improvements in air quality and decarbonization.

Of note, climate strategies are often framed primarily around long-term CO_2_ mitigation [55]. While indispensable, such approaches are constrained by psychological distance and temporal discounting, which diminish their capacity to deliver perceptible near-term benefits and reinforce the perception of climate action as a deferred challenge [56]. Short-lived climate pollutants such as BC can help bridge this gap, as their mitigation generates immediate gains for both climate and air quality that complement long-term CO_2_ mitigation strategies [57]. In the transportation sector, this linkage is particularly salient, as high-emitting diesel vehicles remain major sources of both CO_2_ and BC [58,59]. Our results show that, alongside structural measures, traditional controls (such as *Strengthen Standard and Fuel Quality* and *Phase Out Outdated Vehicle*) achieved substantial BC reductions. These reductions delivered measurable near-term climate benefits in parallel with air quality improvements, thereby building confidence in broader greenhouse gas mitigation efforts. Explicitly integrating BC into transport policy design can therefore yield visible and tangible benefits, providing policymakers with a sense of progress and efficacy and creating a positive feedback loop that sustains momentum for long-term decarbonization [60]. Yet despite this potential, BC has been explicitly recognized in the Nationally Determined Contributions (NDCs) of only a handful of countries [57,61], underscoring the need to elevate its role within both sectoral and national strategies to translate near-term gains into sustained climate progress. More broadly, in sectors and regions where SLCP abatement is feasible, jointly targeting SLCPs and long-lived greenhouse gases offers a pathway to achieve meaningful near-term synergies while reinforcing the foundations for enduring decarbonization.

Beyond carbon and air pollution co-control, the Synergy Index provides a clear way to look at the challenges of multi-objective environmental governance. Environmental governance often tries to meet several goals through policy packages that create different kinds of synergies. By bringing together the impacts of diverse policies into one framework, it shows which portfolios bring joint benefits and which mainly serve a single goal. This perspective supports the design of policy packages that improve overall governance, and it also makes it possible to find measures that gave co-benefits even in a policy history mostly shaped by single-objective policies. As with any composite indicator, however, its application entails methodological uncertainties, notably the choice of normalization baseline and the weighting of health versus climate metrics. In this study, both components were normalized against 2015 totals of CO_2_e emissions and premature deaths, with robustness checks conducted using 2020 totals as an alternative baseline (Table S13). Additional sensitivity analyses varied the health weight from 0.1 to 0.9 in increments of 0.1 (Fig. S5), confirming that the results derived from the Synergy Index remain stable and robust across alternative specifications.

In conclusion, while traditional environmental policies have driven substantial progress in the past, their effectiveness may wane in the future. Strengthening structural transition policies provide a pathway to achieving stronger and more stable synergies between climate and public health benefits, ensuring sustainable progress in carbon-pollution co-control within China’s on-road transportation sector. The dual challenge of reconciling air-quality targets with carbon peaking and neutrality is not unique to China, as rapidly motorizing economies such as India face similar pressures with rising vehicle demand [62]. China’s staged approach, which first delivers early synergies mainly through traditional policies and is progressively complemented by more specific structural transformations to secure lasting benefits, provides a reference for countries at comparable stages of development and broader lessons for global carbon air-pollution co-control.

## METHODS

An integrated analytical framework is developed to estimate and compare the synergetic effectiveness of policies in China’s on-road transportation sector over two periods (Period I: 2010–2015 and Period II: 2015–2020) (Fig. S3). Subperiods are defined by the Twelfth and Thirteenth Five-Year Plans (Fig. 1g), which anchor the analysis within China’s institutional governance framework and ensure that policy impacts are captured without bias from short-term fluctuations. This framework leverages detailed emission inventories for air pollutants and CO_2_ to establish baseline emissions and create policy-specific scenarios for impact assessment. Meanwhile, three counterfactual scenarios for 2020 are developed, incorporating stronger structural transitions in vehicle fleets and transportation structure, as outlined in the Fourteenth Five-Year Plan (14th FYP; 2021–2025), to validate the efficacy of the policy. A Synergy Index, integrating both climate and health metrics, is introduced to quantify the synergetic benefits of the policies and explore pathways for achieving carbon-pollution co-control. Specifically, climate impacts are quantified in CO_2_e emission reductions (derived from CO_2_ and BC reductions), while health benefits, measured as avoided premature deaths attributed to changes in PM_2.5_ and O_3_ concentrations (simulated using the WRF-CAMx modeling system), are evaluated using the GEMM and the GBD dataset. Detailed descriptions of the methods, scenarios, and models used in this study are provided in the Supplementary Information.

### Scenario design

In China, a suite of environmental policies targeting on-road transportation has been implemented to reduce air pollution and CO_2_ emissions. As detailed in the Supplementary Information, these policies include *Strengthen Standard and Fuel Quality (SEQ), Phase Out Outdated Vehicle (POV), Improve Fuel Efficiency (IFE), Optimize Transportation Structure (OTS)*, and *Promote New Energy Vehicle (PNV)*. For each policy, we construct a policy-specific scenario in which the respective actions are not implemented or updated during the periods 2010–2015 (Period I) and 2015–2020 (Period II). Emission reductions for key pollutants (NO*_x_*, VOCs, primary PM_2.5_, BC, and CO) and CO_2_ attributable to each control policy are then quantified using an evaluation approach that integrates scenario analysis with a detailed emission inventory developed for the designated subperiods. In policy-specific scenarios, only the parameters directly influenced by the targeted policy are adjusted. For each period, a NoCtrl scenario that excludes all control policies is also established, and the differences between the BASE and NoCtrl scenarios define the total emission reductions attributable to the combined implementation of all policies. The total difference serves as the constraint for evaluating individual policy contributions, ensuring that their aggregation remains consistent with the overall reductions. A detailed description of the scenario design is provided in Note S2 and Table S4, and the scenarios for evaluating policy-specific effectiveness are summarized in the POLICY scenario group in Table S5.

According to the 14th Five-Year Plan, substantial updates are introduced in transportation structure optimization, vehicle electrification targets, and the phase-out of China Three vehicles, whereas other conventional policies have not been associated with comparable new milestones during this period. Building on these changes, as well as the potential of pollution and GHG synergetic control from these structural transition measures suggested by our historical analysis, three counterfactual enhanced structural transition scenarios for 2020 are further proposed in this study to demonstrate the efficacy of stronger structural policies in further improving synergies in China’s on-road carbon-pollution co-control. In these scenarios, the structural transition targets of the 14th FYP are assumed to be achieved ahead of schedule in 2020, while other socioeconomic and policy factors are assumed to remain unchanged at the 2020 levels. The *Strengthen Vehicle Fleet Structure Transition (FleTran)* scenario assumes that the on-road sector meets the phase-out target for conventional vehicles and achieves the electric vehicle (EV) penetration rate for new registrations by 2020, as specified in the 14th FYP. The *Strengthen Transportation Structure Transition (TraTran)* scenario assumes that the fractions of on-road passenger and freight turnover in 2020 decrease to the levels outlined in the 14th FYP. The *Combined Structure Transition (StrTran)* scenario integrates both vehicle fleet and transportation structure adjustments. The resulting CO_2_e reductions, air quality improvements, public health protection, and Synergy Index values are estimated using the same methods applied to the actual five control policies. Table S6 summarizes the key assumptions and design features of counterfactual scenarios. Full details are provided in Note S7.

### Evaluation of measure-specific impacts

The climate impacts of emission control policies are quantified based on the reductions in CO_2_ and BC emissions resulting from on-road environmental policies. BC is selected as the SLCP in this analysis because the on-road transportation sector is a major anthropogenic source and BC plays a well-documented role in climate-air quality interactions [7,58,63–65]. Given our focus on the short-term discounted effects of policy implementation, we adopt a 20-year global warming potential (GWP_20_) to convert BC emissions into CO_2_ equivalents [56]. Specifically, we use a GWP_20_ value of 1189 (standard deviation: 854.39) for BC in China, as reported in previous studies [10].

The air quality impacts of emission control policies are quantified by examining changes in population-weighted PM_2.5_ (PWPM_2.5_) and population-weighted maximum daily 8-hour mean O_3_ (PW MDA8 O_3_) concentrations resulting from on-road emission reductions. To minimize the influence of non-linear relationships between emissions and modeled concentrations, a NoCtrl scenario was constructed for each estimation year in which none of the five on-road emission control policies were implemented or updated (Table S5). The concentration difference between BASE and NoCtrl defines the total air quality improvement attributable to the simultaneous implementation of all policies. When the sum of policy-specific effects deviated from the total air-quality improvement due to non-linear chemistry, the individual effects were proportionally rescaled so that their combined impact matched the overall improvement (Equation S17). These normalized contributions were subsequently used to attribute changes in PM_2.5_ and summer MDA8 O_3_ to on-road emission controls. The contributions of on-road transportation emissions to surface PM_2.5_ and O_3_ concentrations are performed using the Particulate Source Apportionment Technology (PSAT) and Ozone Source Apportionment Technology (OSAT) modules of the Comprehensive Air Quality Model with Extensions (CAMx) version 6.20 [33], driven by the Weather Research and Forecasting model (WRF) version 3.9 [66]. The WRF-CAMx model demonstrates good and robust performance in simulating PM_2.5_ and O_3_, and the simulated concentrations serve as input for subsequent health impact assessments (Tables S7–S12 and Fig. S4). Details on simulations of air quality changes attributable to control policies, air quality simulation methods, model configurations, and scenario groups are provided in the Supplementary Information.

The health impacts of emission control policies are assessed by calculating the difference in premature deaths associated with long-term PM_2.5_ and O_3_ exposure attributable to on-road emission reductions. The GEMM model is employed to estimate the number of PM_2.5_-attributable premature deaths avoided by each policy for non-communicable diseases (NCDs) and lower respiratory infections (LRIs) [34]. For estimating the number of O_3_-attributable premature deaths avoided, we adopt the method documented in the GBD study [67], which utilizes data from various cohort studies worldwide to estimate O_3_-related non-accidental mortality attributable to chronic obstructive pulmonary disease (COPD). The total avoided premature deaths resulting from the implementation of the five control policies are calculated as the difference between the NoCtrl and BASE scenarios. Policy-specific health benefits were then attributed using a direct proportion approach (Equation S23) [68], which allocates avoided premature deaths in proportion to each policy’s contribution to the reductions in PM_2.5_ and summer MDA8 O_3_ concentrations. This method is validated by the GBD MAPS study [68] and has been widely applied in subsequent research [69–71]. More details can be found in the Supplementary Information.

### Evaluation of measure-specific Synergy Index

The Synergy Index provides a unified framework for assessing both synergies and trade-offs between protecting public health and reducing greenhouse gas emissions, thereby capturing the comprehensive impacts of environmental policies and tracking their evolving synergistic effects across different implementation periods. Given that PM_2.5_ and O_3_ have distinct atmospheric formation mechanisms [72,73], combining their concentrations into a single dimension would lack physical interpretability and risk misrepresenting the actual burden of exposure. Therefore, air quality is not included as an independent dimension in the Synergy Index. Instead, its contribution is represented through the health metric, which is consistent with the primary aim of air pollution governance to improve population health [74]. Two key metrics—relative changes in health and climate impacts—are employed, and their synergies are quantified using the Coupling Coordination Degree (CCD) model [75,76], which has proven effective in analyzing interactions among multiple systems [77–79]. In this approach, relative changes are calculated as the ratio of policy-specific impacts (e.g. avoided premature deaths or CO_2_e emission reductions) to the fixed total contributions of on-road transportation in 2015. Specifically, for each policy, the health metric is defined as the ratio of policy-specific health impacts (i.e. avoided premature deaths) to the total premature deaths attributable to PM_2.5_ and O_3_ exposure related to on-road transportation in 2015 (${M}_{2015}$). Similarly, the climate metric is defined as the ratio of policy-specific climate impacts (i.e. CO_2_e emission reductions) to CO_2_e emissions from on-road transportation in 2015 ($Emi{s}_{C{O}_2e,2015}$). The normalization to the 2015 totals of CO_2_e emissions and premature deaths provides a fixed reference point for all calculations and ensures direct comparability of both sub-metrics, as well as the overall index, across policies and periods. The policy-specific health metric ($H{M}_{\textit{POLICY}}$) and climate metric ($C{M}_{\textit{POLICY}}$) in year *y* can be estimated as follows:


(1) \begin{eqnarray*}
H{M}_{\textit{POLICY},y} = \frac{{\Delta {M}_{\textit{POLICY},y}}}{{{M}_{2015}}},
\end{eqnarray*}

(2) \begin{eqnarray*}
C{M}_{\textit{POLICY},y} = \frac{{\Delta Emi{s}_{C{O}_2e,\textit{POLICY},y}}}{{Emi{s}_{C{O}_2e,2015}}},
\end{eqnarray*}

where $\Delta {M}_{\textit{POLICY}}$ represents the policy-specific premature death changes attributable to air pollution exposure caused by on-road emissions; $\Delta Emi{s}_{C{O}_2e,\textit{POLICY}}$ is the policy-specific CO_2_e emission reductions. The health metric ($H{M}_{TOL}$) and climate metric ($C{M}_{T{\mathrm{OL}}}$) of all policies combined are calculated as follows:


(3) \begin{eqnarray*}
H{M}_{TOL,y} = \frac{{\Delta {M}_y}}{{{M}_{2015}}},
\end{eqnarray*}

(4) \begin{eqnarray*}
C{M}_{T{\mathrm{OL}},y} = \frac{{\Delta Emi{s}_{C{O}_2e,y}}}{{Emi{s}_{C{O}_2e,2015}}},
\end{eqnarray*}

where $\Delta Emi{s}_{C{O}_2e}$ is the total CO_2_e emission reductions attributable to all policy combinations; $\Delta {M}_y$ denotes the total avoided premature deaths attributable to all policy combinations. The Synergy Index ($SynI$) is further calculated as follows [75,76]:


(5) \begin{eqnarray*}
\textit{SynI} = \sqrt {\frac{{2\sqrt {HM \times CM} }}{{HM + CM}} \times \left( {\alpha HM + \beta CM} \right)},
\end{eqnarray*}

where $\alpha $ and $\beta $ are the weights of subsystems and $\alpha + \beta = 1$. Due to the equal importance of air quality-associated health and climate, $\alpha $ and $\beta $ are both 0.5. The values for $HM$, $CM$, and $SynI$ range from 0 to 1, with higher values indicating better performance. In cases where a policy results in adverse effects or no impacts on either health or climate, the SynI is assigned a value of 0. Detailed descriptions of the methods and associated uncertainty analyses are provided in the Supplementary Information.

## Supplementary Material

nwaf422_Supplemental_File

## Data Availability

The MEIC emission inventory is available from http://meicmodel.org. The dataset generated during this study can be accessed from https://doi.org/10.6084/m9.figshare.30318691.v1.

## CODE AVAILABILITY

The code of the WRF model is available at https://www2.mmm.ucar.edu/wrf. The code of the CAMx model is available at https://www.camx.com/download. The codes used for analyzing data are available upon reasonable request from the corresponding author.
