# Supplementary material for: Weakening synergies in carbon and air pollution co-control necessitate robust structural transitions in China’s transportation sector
Source: Natl Sci Rev. 2025 Sep 27;12(11):nwaf422. doi: 10.1093/nsr/nwaf422 (PMC12604017; doi:10.1093/nsr/nwaf422)
Supplement: nwaf422_Supplemental_File [file nwaf422_supplemental_file.docx]

Supplementary Information for
Weakening synergies in carbon and air pollution co-control necessitate robust structural transitions in China’s transportation sector

Zhulin Qi^1,2,^***^†^***, Yixuan Zheng^2,*,^***^†^***, Wenxin Cao^2,3^, Yuxi Liu^2^, Xin Li^4^, Yueying Fei^4^, Shigan Liu^5^, Fangming Jiang^1,2^, Chuchu Chen^2^, Yueyi Feng^2^, Zhang Wen^2^, Xuying Wang^2^, Yu Lei^2^, Zhibin Wang^1^, Gang Yan^2,*^ and Jinnan Wang^2,1,*^

^1^College of Environmental & Resource Sciences, Zhejiang University, Hangzhou 310058, China.

^2^Key Laboratory of Environmental Pollution and Greenhouse Gases Co-control, Ministry of Ecology and Environment, Chinese Academy of Environmental Planning, Beijing 100041, China.

^3^College of New Energy and Environment, Jilin University, Changchun, 130012, China.

^4^Department of Environmental Science and Engineering, Beijing Technology and Business University, Beijing 100048, China.

^5^Department of Earth System Science, Ministry of Education Key Laboratory for Earth System Modeling, Institute for Global Change Studies, Tsinghua University, Beijing 100084, China.

*Corresponding authors: Yixuan Zheng (zhengyx@caep.org.cn), Gang Yan (yangang@caep.org.cn), and Jinnan Wang (wangjn1962@126.com).

***^†^***These authors contributed equally to this work.

**Table of Contents**

| Number | Contents | Page |
| --- | --- | --- |
| 1 | Summary of environmental policies targeting China’s on-road transportation sector over 2010-2020 | 4 |
| 2 | Estimation of air pollution and GHG emission reductions attributable to control policies | 9 |
| 3 | Baseline air quality simulations and evaluations | 15 |
| 4 | Simulations of air quality changes attributable to emission control | 21 |
| 5 | Simulations of avoided premature deaths attributable to control policies | 23 |
| 6 | Development of the Synergy Index | 26 |
| 7 | Design of enhanced structural adjustment scenarios for 2020 | 28 |
| 8 | Uncertainties and limitations | 31 |
| 9 | Supplementary Figure 1 | 35 |
| 10 | Supplementary Figure 2 | 36 |
| 11 | Supplementary Figure 3 | 37 |
| 12 | Supplementary Figure 4 | 38 |

**Table of Contents (continued)**

| Number | Contents | Page |
| --- | --- | --- |
| 13 | Supplementary Figure 5 | 39 |
| 14 | Supplementary Table 1 | 40 |
| 15 | Supplementary Table 2 | 41 |
| 16 | Supplementary Table 3 | 42 |
| 17 | Supplementary Table 4 | 44 |
| 18 | Supplementary Table 5 | 45 |
| 19 | Supplementary Table 6 | 47 |
| 20 | Supplementary Table 7 | 48 |
| 21 | Supplementary Table 8 | 49 |
| 22 | Supplementary Table 9 | 50 |
| 23 | Supplementary Table 10 | 51 |
| 24 | Supplementary Table 11 | 52 |
| 25 | Supplementary Table 12 | 53 |
| 26 | Supplementary Table 13 | 54 |
| 27 | References | 55 |

*Supplementary Methods:*

**Supplementary Note 1: Summary of environmental policies targeting China’s on-road transportation sector over 2010-2020**

To address air pollution and climate issues associated with on-road transportation, the Chinese government has implemented a set of environmental policies from 2010 to 2020 to control both air pollution and carbon emissions, which fall into five key categories: *Strengthen Standard and Fuel Quality*, *Phase Out Outdated Vehicle*, *Improve Fuel Efficiency*, *Optimize Transportation Structure*, and *Promote New Energy Vehicle*. A detailed description of these policies is provided below, with a brief overview of actions shown in Fig. 1g.

**Summary of Socioeconomic Trends and Policy Drivers.** Figure 1 shows the trends in key socioeconomic and policy drivers affecting emissions from China’s on-road transportation sector. During the 2010s, China’s total vehicle fleet expanded at an annual rate of 6.2%, increasing from 178.9 million to 325.4 million (Fig. 1a) [1]. Notably, among all vehicle categories, light-duty passenger vehicles (LDPVs) and heavy-duty trucks (HDTs)—identified as the primary GHG and air pollution co-emitters (Extended Data Fig. 1)—tripled in number (Fig. 1a) [1]. Although such growth would typically exacerbate emissions, the enforcement of stricter standards and concurrent structural transitions may partially counterbalance these effects (Fig. 1g). The implementation of mandatory standards—bolstered by technological advancements—has reduced emission intensity and enhanced energy efficiency. The *Strengthen Standard and Fuel Quality* policy has driven three successive upgrades in air pollutant emission standards for both LDPVs (from China 3 to China 6) and HDTs (from China III to China VI) within the same period (Fig. 1g) [2, 3]. Furthermore, the *Phase Out Outdated Vehicle* initiative was introduced to replace old cars and trucks with newer, more efficient models. Under the two phases of the national Clean Air Action Plan (2013-2017, and 2018-2020), over 26 million outdated vehicles were removed from the roads (Fig. 1g) [2, 3]. By 2020, over 70% of the vehicles complied with China four (4/Ⅳ) or stricter standards (Fig. 1b), underscoring the country’s resolute commitment to air quality improvement [4]. Moreover, over a ten‐year span, the *Improve Fuel Efficiency* policy are expected to exert a 35.8% reduction in the corporate average fuel consumption (CAFC) for LDPVs [5] (Fig. 1c), suggesting a proportional decrease in CO_2_ emission intensity. Complementing these standard enforcement actions, the *Promote New Energy Vehicle* and *Optimize Transportation Structure* are designed to support the transition of the on-road transportation structure to cleaner fuel vehicles and more efficient transportation modes. Specifically, the *Optimize Transportation Structure* strategy further reduced vehicle reliance by encouraging alternative modes for passenger and freight transport, as evidenced by the decline in the on-road passenger turnover fraction from 63.0% to 35.9% and the on-road freight turnover fraction from 33.2% to 30.6% over the decade (fig. 1d,e) [1]. Additionally, the post-2018 “modal shift from road to rail and waterways” (RRW) measure corresponded with a significant drop in on-road freight turnover [1]. Meanwhile, as a major effort to decarbonize the vehicle fleet, the *Promote New Energy Vehicle* policy, has spurred exponential, albeit modest, growth in electric vehicle (EV) adoption between 2010 and 2020 (Fig. 1f) [6].

**Strengthen Standard and Fuel Quality.** The foundation of China’s air pollution control system for on-road transportation is built upon progressively stricter emission standards and the adoption of cleaner fuel qualities. Between 2010 and 2020, the nation undertook three major revisions of its emission standards for both gasoline and diesel vehicles, evolving from the China 3/Ⅲ to the current China 6/Ⅵ (Supplementary Table 2 and Fig. 1g). These updated standards were implemented in phases. In 2011, the government introduced the China 4 standard for light-duty passenger vehicles (LDPVs) (GB 18352.3-2005), and two years later, extended China IV to heavy-duty trucks (HDTs) (GB 17691-2005). To further mitigate air pollution—particularly by reducing NO*_x_* emissions from HDTs and VOC emissions from LDPVs—China adopted even stricter standards. The China 5/Ⅴ standard was implemented in 2017 (GB 18352.3-2013 and GB 17691-2005), followed by the China 6/Ⅵ standard in 2020 (GB 18352.3-2016 and GB 17691-2018) for both gasoline and diesel vehicles. As a consequence, the implementation of stricter emission standards yielded significant reductions in baseline emission factors: for HDTs, NO*_x_* emission factors decreased by approximately 30% (to 5.55 g/km) from 2010 to 2015 and by a further 25% (to 0.06 g/km) from 2015 to 2020; similarly, VOCs emission factors for LDPVs were reduced by roughly 60.7% (to 0.075 g/km) from 2010 to 2015 and by an additional 25.3% (to 0.056 g/km) from 2015 to 2020 [7, 8]. Notably, some developed regions (e.g., Beijing and Shanghai) adopted emission standards earlier than required at the national level. In addition, the policy framework for fuel quality improvement in China during 2010-2020 was devised to align fuel standards with increasingly stringent vehicle emission regulations—a process that, unlike in many developed countries, initially lagged behind emission standard upgrades. Recognizing this discrepancy, the Chinese government mandated nationwide enhancements in fuel quality to complement the tightening of vehicle emission standards. Accordingly, the China 4 gasoline technical standard (GB 17930-2011) was issued in 2014, followed by the China IV diesel technical standard (GB 19147-2013) in 2015. In 2017, the framework was further updated with the introduction of the China 5 gasoline and China V diesel technical standards (GB 19147-2013 and GB 17930-2013). As a result, the maximum sulfur content of diesel fuel had to be reduced from 2000 ppm to 50 ppm between 2010 and 2015, and further reduced from 50 ppm to 10 ppm between 2015 and 2020. Similarly, the maximum sulfur content of gasoline had to be reduced by 66.7% to 50 ppm between 2010 and 2015, and further reduced by 80% to 10 ppm between 2015 and 2020.

**Phase Out Outdated Vehicle.** Since the implementation of the two phases of Clean Air Actions in 2013, a phased approach has been adopted to eliminate outdated vehicles that do not meet specific emission standards [2, 3]. Under the Air Pollution Prevention and Control Action Plan (2013-2017), more than 20 million vehicles were removed from the fleet, primarily targeting gasoline vehicles that did not meet the China 1 standard and diesel vehicles that failed to meet the China III [2]. Building on these initial efforts, the subsequent Three-Year Action Plan for Winning the Blue Sky Defense Battle (2018-2020) further intensified measures, specifically targeting diesel vehicles. Under this plan, nearly 6 million additional vehicles were removed from the fleet as the focus shifted to the early retirement of diesel vehicles that did not meet stricter emission standards [3].

**Improve Fuel Efficiency.** The Fuel Consumption Regulation (FCR) serves as a cornerstone of China’s strategic commitment to improving fuel efficiency and controlling CO_2_ emissions. Between 2010 and 2020, the regulatory framework underwent systematic revisions. For light-duty passenger vehicles (LDPVs), Phase III of the FCR was implemented in 2012, introducing Corporate Average Fuel Consumption (CAFC) as a regulatory upper limit for manufacturers and setting a target of 6.9 L/100 km by 2015 (GB 27999-2011). Building on this progress, Phase IV, implemented in 2016, established a more ambitious target of 5.0 L/100 km by 2020 (GB 19578-2014; GB 27999-2014). For heavy-duty vehicles (HDVs), fuel efficiency regulation began with the industry standard QC/T 924-2011, implemented in 2012 (Phase I). Recognizing the need for stricter requirements, the Ministry of Industry and Information Technology (MIIT) introduced the first national standard, Phase II, in 2014 (GB 30510-2014; effective in 2015), followed by Phase III in 2019 (GB 30510-2018).

**Optimize Transportation Structure.** Optimizing the transportation structure is a primary measure aimed at adjusting both passenger and freight transportation systems to reduce emissions from on-road vehicles. From 2010 to 2020, China rapidly expanded its public transportation infrastructure, particularly by shifting medium- and long-distance travel from road-based transportation to more efficient modes, such as high-speed railways. For instance, the percentage of passengers opting for non-road long-distance travel increased from 0.7% to 16.7% between 2010 and 2015, then further rose to 26.9% during 2015-2020 [1]. In addition, in recent years, the Chinese government has actively promoted the optimization of freight transportation structures, particularly for bulk cargo, through initiatives like the “modal shift from road to rail and waterways” (RRW). Guided by the Three-Year Action Plan for Promoting Transportation Structure Adjustment since 2018, China’s total non-road freight turnover increased by 6.25%, from 12.8 × 10^12^ t·km to 13.6 × 10^12^ t·km, between 2018 and 2020 [9].

**Promote New Energy Vehicle.** The adoption of new energy sources (e.g., electricity and natural gas) in vehicle fleets emerged as a primary strategy for on-road transportation between 2010 and 2020, aiming to reduce dependence on traditional fossil fuels and the associated anthropogenic emissions. In China, initial efforts focused on integrating natural gas into vehicle fleets, particularly taxis and public buses [10]. However, a significant proportion of these alternative fuel vehicles were essentially modified versions of conventional vehicles, and their estimated environmental benefits fell short of initial expectations [11-13]. In response, electric vehicles (EVs) were introduced in China, such as the demonstration program “Ten Cities and Thousand Vehicles” conducted from 2008 to 2014 [14]. Subsequently, in 2012, the central government set an ambitious target of producing and selling up to 5 million EVs by 2020 [13]. Between 2017 and 2020, the population of new energy vehicles in China surged by 226.7%, rising from 1.5 million to 4.9 million, with over 80% being electric vehicles [9].

**Supplementary Note 2: Estimation of air pollution and GHG emission reductions attributable to control policies**

An analytical framework was developed to estimate air pollution and GHG emission reductions attributable to control policies in China’s on-road transportation sector. The study period was divided into two subperiods (2010-2015 and 2015-2020), aligned with the Twelfth and Thirteenth Five-Year Plans, which provide the overarching institutional frameworks guiding national strategies on environmental protection and climate mitigation. Major air pollution and carbon-control policies were fully incorporated. For instance, the Clean Air Action Plans, which represent important milestones in air quality management, are embedded within and implemented under the guidance of the Five-Year Plans as specific policy instruments. For each subperiod, we constructed a set of policy-specific scenarios corresponding to the five key policies described in Supplementary Note 1 and shown in Fig. 1g, in order to quantify the emission reductions achieved by on-road transportation policies. In these scenarios, we assume that the targeted policy was either never implemented or was not reinforced during the subperiod. When compiling the emission inventories for policy-specific scenarios, only the parameters directly affected by the targeted policy were adjusted. This design explicitly avoids potential overlaps among policies. For example, although stricter emission standards and the phase-out of older vehicles may both affect fleet structure, they are represented through distinct parameters: emission standards lower the emission factors of newly registered vehicles, whereas phase-out policies accelerate the reduction of older, high-emitting vehicles. By parameterizing these interventions separately, the framework prevents double counting and ensures that their effects are not conflated. Additionally, a NoCtrl scenario, in which no control policies are implemented, was established for each period. The difference between the BASE and NoCtrl scenarios defines the total emission reductions attributable to the simultaneous implementation of all policies. This total serves as a constraint on policy attribution, ensuring that the combined contributions from individual policy scenarios do not exceed the overall benchmark. Provincial-level emission reductions for major air pollutants (NO*_x_*, VOCs, primary PM_2.5_, CO, and BC) and CO_2_ under each policy-specific scenario were quantified using a bottom-up approach that incorporates key assumptions, which can be calculated as follows:

| ${\Delta Emis}_{i,p,y,m}=\sum_{t} \sum_{f} \sum_{s} ({\eta^{'}}_{t,p,m}\times{{VP}^{'}}_{t,f,s,p,y,m}\times{{EF}^{'}}_{i,t,f,s,p,y,m}\times{VKT}_{t,f,s,p,y,m})-{Emis}_{i,p,y}$ | (S1) |
| --- | --- |

where $\Delta Emis$ represents the emission reductions of each policy; $m$ represents the emission policies summarized in this study; $i$ represents air pollutants or CO_2_; ${{VP}^{'}}_{t,f,s}$ is the estimated vehicle population under the policy-specific scenario, categorized by vehicle type $t$, fuel type $f$, and emission standard $s$; ${EF}^{'}$ is the estimated emission factors under the policy-specific scenario; $VKT$ is the annual fleet average vehicle kilometers traveled; $\eta^{'}$ denotes the estimated on-road transportation demand under the policy-specific scenario; $Emis$ refers to the baseline emission estimates, which is documented in the previous paper [4]; subscript $p$ and $y$ represent the province and target year. The detailed estimations for the linked vehicle population (${VP}^{'}$), emission factors (${EF}^{'}$), and on-road transportation demand ($\eta^{'}$) for each of the five policy-specific scenarios are documented below.

**Strengthen Standard and Fuel Quality.** The implementation of stringent vehicle emission standards necessitates advanced end-of-pipe treatment technologies in new vehicles. Fig. 1g and Supplementary Table 2 summarize the timeline for introducing specific emission standards across various vehicle types. As these new standards are implemented, the emission efficiency (${{EF}^{'}}_{s}$ in Eq. (S1)) of the vehicle fleet substantially improves (Fig. 1b). Under the policy-specific scenario, we assume that no additional enhancements in end-of-pipe treatment occur after the base year (i.e., 2010 for Period I and 2015 for Period II). Accordingly, vehicles registered in any given subperiod are assumed to maintain the emission factor level established in the corresponding base year. For example, in Period I under the policy-specific scenario, if the government does not upgrade the emission standard for light-duty passenger vehicles (LDPVs) to China 4, then newly registered LDPVs are assumed to adhere to the base emission factors of China 3. The adoption of cleaner fuel qualities is primarily realized through improvements in emission factors *(*${EF}^{'}$ in Eq. (S1)). Specifically, cleaner fuels lead to a reduction in the fuel adjustment factors applied to the base emission factors, thereby lowering pollutant emissions per unit of travel distance [7]. Under the policy-specific scenario, we assume that fuel quality remains constant at the base-year level, implying that no advancements in fuel formulation or regulatory standards are introduced.

**Phase Out Outdated Vehicle.** Under the policy-specific scenario, we assume that no additional phase-out actions take place after the base year (${{VP}^{'}}_{s}$ in Eq. (S1)). Consequently, the retirement rate of outdated vehicles remains at the natural scrappage rate of the base year in each subsequent subperiod, following the methodology of Zhou et al. [15]. Furthermore, the overall vehicle stock is assumed to remain constant, aligning with baseline emission estimates for the target year. The natural scrappage rate of vehicles is:

| $\sigma_{y0,p,t}=\frac{{VP}_{y0-1,p,t}+{NV}_{y0,p,t}-{VP}_{y0,p,t}}{{VP}_{y0,p,t}}$ | (S2) |
| --- | --- |

where $\sigma_{p,t}$ is the natural scrappage rate of vehicle type $t$ in province $p$ during the subperiod; $y0$ represents the base year, that is, 2010 for Period Ⅰ and 2015 for Period Ⅱ; $y$ is the target year; and $NV$ denotes the number of newly registered vehicles; $VP$ represents the actual vehicle population. Then, the estimated number of newly registered vehicles in the target year $y$ under the policy-specific scenario (${{NV}^{'}}_{y}$) can be expressed as:

| ${OV}_{y0,p,t}\times{{(1-\sigma}_{y0,p,t})}^{y-y0}+{{NV}^{'}}_{y,p,t}={VP}_{y,p,t}$ | (S3) |
| --- | --- |

where $OV$ represents the number of outdated vehicles in the base year.

**Improve Fuel Efficiency.** Improvements in fuel efficiency are primarily achieved through the implementation of stringent new fuel consumption regulations. These regulations are designed to reduce the average fuel consumption of vehicles, thereby lowering air pollution and CO_2_ emissions over the same travel distance. To account for the impacts of improved fuel efficiency on emissions, our methodological assumption is that enhanced fuel efficiency leads to a reduction in fuel consumption per unit of distance traveled, which, in turn, results in a decrease in emissions produced by fuel combustion. Under the policy-specific scenario, we define the emission factor per unit distance traveled (${EF}^{'}$ in Eq. (S1)) as being adjusted by a factor $\omega$, which is calculated as the ratio of the base-year fuel efficiency (${FE}_{y0}$) to the target-year fuel efficiency (${FE}_{y}$):

| ${{EF}^{'}}_{i,t,f,s,p,y}=\omega\times{EF}_{i,t,f,s,p,y}$  $=\frac{{FE}_{i,t,f,s,p,y0}}{{FE}_{i,t,f,s,p,y}}\times{EF}_{i,t,f,s,p,y}$ | (S4) |
| --- | --- |

where ${EF}_{i,t,f,s,p,y}$ specifically represents the baseline emission factor of air pollutants or CO_2_ in province $p$ in target year $y$, categorized by vehicle type $t$, fuel type $f$, and emission standard $s$.

**Optimize Transportation Structure.** This policy aims to replace conventional on-road transportation modes with more energy-efficient alternatives, thereby reducing on-road transportation demand ($\eta^{'}$ in Eq. (S1)). As shown in Fig. 1d-e, the fractions of on-road passenger and freight turnover decreased to 35.9% and 30.6%, respectively, between 2010 and 2020. Although previous studies have noted that shifts in transportation modes could affect vehicle activity and alter VKT [16-18], it is important to recognize that changes in VKT are not entirely independent but are highly influenced by vehicle stock levels [19]. This indicates that variations in VKT alone cannot fully explain improvements in the transportation structure. To address this complexity, we introduce an adjustment factor, $\eta$, to reflect on-road transportation demand changes under the policy-specific scenario. This factor is estimated directly from interannual changes in the fractions of transportation turnover at the provincial level. Under the policy-specific scenario, we assume that no further transitions in the transportation structure occur beyond the base year. Specifically, we assume that no on-road passenger transportation transitions occur after 2010 for Period I and after 2015 for Period II, and no on-road freight transportation transitions occur after 2018 for Period II. We therefore project that the fractions of on-road transportation turnover would remain at the level of the base year. In addition, in cases where the on-road transportation turnover proportion exhibits a growth trend in province $p$, we assume that no improvements in the transportation structure have occurred in that province. Therefore, such provinces are excluded from the policy-specific estimation. The adjustment factor $\eta$ can be calculated as:

| ${ratio}_{p,sub}=\frac{\frac{T_{on,y0,p,sub}}{T_{tr,y0,p,sub}}\times T_{tr,y,p,sub}}{T_{on,y,p,sub}}$ | (S5) |
| --- | --- |
| $\eta_{p,sub}=\left\{ \begin{aligned} {ratio}_{p,sub}, &if {ratio}_{p,sub}>1 \\ 1, &if {ratio}_{p,sub}\leq1 \end{aligned} \right.$ | (S6) |

where $\eta_{sub}$ represents the adjustment factor for vehicles in subsector $sub$ (as defined in Supplementary Table 1); $T_{on}$ represents the passenger or freight turnover of on-road transportation;$T_{tr}$ represents the total passenger or freight transportation turnover; $ratio$ represents the comparison between the projected on-road turnover proportion and the actual on-road turnover proportion in the target year $y$.

**Promote New Energy Vehicle.** The implementation of new energy vehicle promotion primarily alters the fuel structure of the vehicle fleet (${{VP}^{'}}_{f}$ in Eq. (S1)) by replacing traditional fuels with electricity and natural gas. The uptake of new energy vehicles in China between 2010 and 2020 was predominantly driven by government incentives and policies, resulting in a low penetration rate among new vehicle sales (Fig. 1f,g). Based on this observation, we posit that the expansion of the new energy vehicle fleet is inherently tied to continued governmental support. Consequently, in our policy-specific scenario, we assume that once governmental promotion ceases after the base year, no additional new energy vehicles will be introduced into the fleet, and the number of new energy vehicles will remain at the base-year level.

**Supplementary Note 3: Baseline air quality simulations and evaluations**

**Modeling configurations.** In this study, the PM_2.5_ and O_3_ concentrations across China for all scenario groups (Supplementary Table 5) for the years 2015 and 2020 are simulated using the Comprehensive Air Quality Model with Extensions (CAMx) v6.20, which is driven by meteorological conditions simulated by the Weather Research and Forecasting (WRF) model version 3.9 (the WRF-CAMx modeling system). The configuration of the WRF-CAMx modeling system adheres to previous studies [2, 20-22], enabling the simulation of meteorological parameters and air pollution concentrations at a horizontal resolution of 27 km × 27 km over China. The Particulate Source Apportionment Technology (PSAT) and the Ozone Source Apportionment Technology (OSAT) of CAMx are applied to attribute the contributions of air pollutant emissions from the on-road sector to PM_2.5_ and O_3_ concentrations at the model grid level. The WRF model is driven by the initial and boundary conditions (ICs and BCs) obtained from the National Centers for Environmental Prediction Final Analysis (NCEP-FNL) reanalysis data. The CAMx model is configured with CB05 as the gas-phase mechanism, CF as the aerosol module, RADM-AQ as the aqueous-phase oxidation, and ZHANG03 to parametrize dry deposition. Boundary and initial conditions for the CAMx simulations are provided by CAM-Chem simulation outputs [23]. To drive the CAMx model, anthropogenic emission inventories are required as inputs. The air pollutant emissions from on-road transportation are derived from our previous study [4], while anthropogenic emissions from other sectors (including industry, power, residential, agriculture, and off-road transportation) for 2015 and 2020 are obtained from the Multi-resolution Emission Inventory of China (MEIC) [24]. Anthropogenic emissions beyond mainland China are collected from the Mosaic Asian Anthropogenic Emission Inventory (MIX) of 2015 [25]. Natural emissions for the CAMx simulations are estimated using the Model of Emissions of Gases and Aerosols from Nature (MEGAN) version 3.1. The MEGAN v3.1 follows the approach outlined by Liu et al [26] and is driven by real-time meteorological conditions from WRF simulations [27]. For all scenario groups in simulated years, PM_2.5_ and O_3_ concentrations attributable to air pollution emissions from on-road transportation are simulated with a one-month spin-up using WRF-CAMx. For PM_2.5_, daily mean concentrations at the model’s bottom layer are extracted to represent surface PM_2.5_. For O_3_, the maximum daily 8-hour average ozone (MDA8 O_3_) during the warm season (April to September) at the bottom layer is defined as surface O_3_.

**Evaluation of the baseline simulation.** The WRF-CAMx modeling system simulates meteorological parameters and surface pollution concentrations for the two examined years (i.e., 2015 and 2020). The baseline model simulations are evaluated against ground-level observations and machine-learning-based data. The statistical parameters considered in this study include the correlation coefficient (R), mean bias (MB), root mean square error (RMSE), normalized mean bias (NMB), normalized mean error (NME), mean fractional bias (MFB), mean fractional error (MFE), and gross error (GE). The equations used to calculate these statistical parameters are as follows:

| $R=\frac{\sum_{i=1}^{n} \left[ \left( {Sim}_{i}-\bar{Sim} \right)\times\left( {Obs}_{i}-\bar{Obs} \right) \right]}{\sqrt{\sum_{i=1}^{n} \left( {Sim}_{i}-\bar{Sim} \right)^{2}}\times\sqrt{\sum_{i=1}^{n} \left( {Obs}_{i}-\bar{Obs} \right)^{2}}}$ | (S7) |
| --- | --- |
| $MB=\frac{1}{n}\times\sum_{i=1}^{n} \left( {Sim}_{i}-{Obs}_{i} \right)$ | (S8) |
| $RMSE=\sqrt{\frac{1}{n}\times\sum_{i=1}^{n} \left( {Sim}_{i}-{Obs}_{i} \right)^{2}}$ | (S9) |
| $NMB=\frac{\sum_{i=1}^{n} \left( {Sim}_{i}-{Obs}_{i} \right)}{\sum_{i=1}^{n} {Obs}_{i}}\times100\%$ | (S10) |
| $NME=\frac{\sum_{i=1}^{n} \left\vert{Sim}_{i}-{Obs}_{i} \right\vert}{\sum_{i=1}^{n} {Obs}_{i}}\times100\%$ | (S11) |
| $NFB=\frac{2}{n}\times\sum_{i=1}^{n} \frac{{Sim}_{i}-{Obs}_{i}}{{Sim}_{i}+{Obs}_{i}}\times100\%$ | (S12) |
| $NFE=\frac{2}{n}\times\sum_{i=1}^{n} \frac{\left\vert{Sim}_{i}-{Obs}_{i} \right\vert}{{Sim}_{i}+{Obs}_{i}}\times100\%$ | (S13) |
| $GE=\frac{1}{n}\times\sum_{i=1}^{n} \left\vert{Sim}_{i}-{Obs}_{i} \right\vert$ | (S14) |

where $Sim$ represents the simulated results by model; $Obs$ donates the observed or the ML-based data; $i$ represents data samples.

Meteorological parameters simulated by the WRF model, including temperature at 2 m, relative humidity at 2 m, wind speed at 10 m, wind direction at 10 m, and daily precipitation, are evaluated using ground-level observations. Specifically, temperature, relative humidity, wind speed, and wind direction are assessed based on ground-level observations obtained from the National Climate Data Center (NCDC, <ftp://ftp.ncdc.noaa.gov/pub/data/noaa/>), while daily precipitation is evaluated using observational data collected from the Global Surface Summary of the Day Data (GSOD, <ftp://ftp.ncdc.noaa.gov/pub/data/gsod/>). Benchmarks derived from various simulations conducted in the eastern USA with 4-12 km grid resolutions on an hourly basis are used for comparison with our evaluation [28]. These benchmarks include MB within ±0.5 K and GE < 2.0 K for temperature, MB within ±0.5 m/s and RMSE < 2 m/s for wind speed, and MB within ±10° and GE < 30° for wind direction. As shown in Supplementary Table 7, the performance of our WRF model, evaluated against observations, is comparable to benchmark metrics. The simulated temperature and relative humidity show good agreement with observations, exhibiting high R values, low mean biases, and low gross error. The MB and GE values for wind speed across all years fall within the benchmark. The MB values for wind direction also meet the benchmark for all years, but the GE values slightly exceed the benchmark, ranging from 48.58 to 48.82. The simulated daily precipitation aligns well with observations, with MB values ranging from 0.16 to 0.22. It is important to recognize that these benchmarks serve as general references for model evaluation rather than strict criteria for determining whether a specific meteorological model application passes or fails. In summary, the WRF model demonstrates acceptable performance in simulating meteorological parameters for further air quality simulations.

The simulated baseline PM_2.5_ and summer MDA8 O_3_ concentrations are evaluated against ground-level PM_2.5_ and O_3_ observations collected from the national monitoring networks established and operated by the China National Environmental Monitoring Center (CNEMC, <http://www.cnemc.cn/en/>). Supplementary Tables 9-10 compare simulated PM_2.5_ and summer MDA8 O_3_ concentrations with surface observations on a daily basis. The simulated PM_2.5_ in China (MFB range from -8.5% to -6.2% and MFE range from 56.5% to 57.6%) are within the criteria limits of MFB (±60%) and MFE (75%) suggested by the United States Environmental Protection Agency (EPA) on a daily basis [29]. The simulated summer MDA8 O_3_ in China (MFB range from 0.4% to 5.1% and MFE range from 23.4% to 34.7%) are within the criteria limits of MFB (±15%) and MFE (35%) suggested by the EPA on a daily basis [29]. The less satisfactory performance of the modeled PM_2.5_ results is observed in western China. Previous studies have also reported the less satisfactory performance of air quality models in accurately simulating PM_2.5_ concentrations in western China due to the dominant influence of windblown dust, whereas anthropogenic emissions—better captured by emission-driven models—play a smaller role compared to eastern China [3, 30-32]. In contrast, the model demonstrates a strong capability in capturing the spatial and temporal variations of PM_2.5_ and summer MDA8 O_3_ concentrations in eastern China, where over 93% of the national population resides [30]. This indicates the effectiveness of the model in simulating PM_2.5_ and summer MDA8 O_3_ concentrations in densely populated and highly polluted areas. Given the sparse population in western China and the primary focus of this study on premature deaths attributable to air pollution exposure, the performance of the model in western China is unlikely to change the conclusion of this study. In general, our model falls within the typical bounds reported in both historical and recent modeling studies.

Gridded annual mean PM_2.5_ and summer MDA8 O_3_ concentrations simulated by the WRF-CAMx modeling system for 2015 and 2020 are also compared with machine-learning-based annual mean PM_2.5_ and seasonal mean MDA8 O_3_ estimates (i.e., ML-based PM_2.5_ and ML-based MDA8 O_3_) collected from Tracking Air Pollution in China dataset (TAP, <http://tapdata.org.cn/>). The TAP dataset is a high-resolution air quality product that synthesizes satellite-derived aerosol optical depth data, outputs from air quality models, and ground-based observations [33-36]. For consistency in comparison, the ML-based PM_2.5_ and MDA8 O_3_ estimates, originally at a 10 km resolution, are spatially projected and regridded to match the 27 km resolution of the CAMx model grid. Supplementary Fig. 4 and Supplementary Tables 11-12 present the evaluation results, comparing simulated PM_2.5_ and MDA8 O_3_ concentrations with the ML-based estimates. As expected, the CAMx model exhibits a systematic underestimation of PM_2.5_ concentrations in western China (grey dots in Supplementary Fig. 4a,b) but demonstrates considerably better performance in simulating PM_2.5_ and MDA8 O_3_ levels in the more populated eastern part of China (blue dots and statistics in blue texts in Supplementary Fig. 4). As discussed previously, the underestimation of PM_2.5_ concentrations in the sparsely populated western part of China, which is likely influenced by natural sources, is not expected to change the conclusions of this study. As shown in Supplementary Tables 11-12 and highlighted by the blue statistics in Supplementary Fig. 4, the simulated PM_2.5_ and MDA8 O_3_ concentrations in eastern China exhibit robust correlations with the ML-based estimates, with R values ranging between 0.80 and 0.90 over the study years. The NMB remain within a consistent range of -15.5% to 15.4% across the investigated years, further supporting the reliability of the CAMx model performance. The evaluation against ML-based datasets indicates that the WRF-CAMx modeling framework effectively captures both spatial and temporal variations in PM_2.5_ and MDA8 O_3_ over eastern China, demonstrating stable performance across the investigated years. Therefore, the simulated PM_2.5_ and MDA8 O_3_ levels can serve as inputs for further health impact assessments.

**Supplementary Note 4: Simulations of air quality changes attributable to emission control**

In our study, we conducted simulations for two distinct policy periods as summarized in Supplementary Table 5. In 2015, the POLICY scenario group includes five policy-specific simulations to evaluate the air quality impacts of five emission control policies. For 2020, the POLICY scenario group similarly includes five policy-specific simulations to assess air quality effects under the prevailing policy status. Additionally, the Counterfactual scenario group introduces three structural transition scenarios for 2020, aimed at quantifying potential further improvements in air quality resulting from structural changes. The simulations of PM_2.5_ and summer MDA8 O_3_ in the POLICY and Counterfactual scenarios utilize meteorological and boundary conditions corresponding to 2015 and 2020, respectively, aligning with those of the BASE scenario for each corresponding year. The high-resolution TAP dataset is then applied to adjust the PM_2.5_ and summer MDA8 O_3_ simulations at the grid level for each scenario, mitigating concentration biases introduced by the WRF-CAMx model. In each POLICY and Counterfactual scenario simulation, the emission reductions introduced by the specific emission control are incorporated into the baseline emissions. The resulting emission inventory is then used to drive the air quality model. The policy-specific impacts of five emission control policies and three structural transition designs on PM_2.5_ and summer MDA8 O_3_ concentrations are calculated as the simulated concentration that are attributable to policy-related emissions derived from the PSAT and OSAT estimates.

To mitigate the nonlinear relationship between emissions and modeled PM_2.5_ as well as summer MDA8 O_3_ concentrations, a NoCtrl scenario is introduced for each subperiod. This NoCtrl scenario represents a case in which none of the five current state environmental policies (that is, *Strengthen Standard and Fuel Quality (SEQ)*, *Phase Out Outdated Vehicle (POV)*, *Improve Fuel Efficiency (IFE)*, *Optimize Transportation Structure (OTS)*, and *Promote New Energy Vehicle (PNV)*) have been implemented. The air quality simulation for the NoCtrl scenarios is conducted by combining baseline emissions with the total emission reductions from these five policies (Supplementary Table 5). The changes in PM_2.5_ and summer MDA8 O_3_ attributable to on-road emissions are then normalized by the difference between the NoCtrl and BASE scenarios. The final policy-specific impacts on population-weighted air pollution concentrations ($\Delta C_{PWPOLICY}$) in year $y$ can be expressed as:

| $\Delta C_{C_{POLICY,j,y}}={(C}_{POLICY,j,y}-C_{BASE,j,y})\times\frac{C_{TAP,j,y}}{C_{BASE,j,y}} \times F_{C_{on-road,j,y}}, POLICY=SEQ,POV,IFE,OTS,PNV$ | (S15) |
| --- | --- |
| $\Delta C_{C_{TOL,j,y}}={(C}_{NoCtrl,j,y}-C_{BASE,j,y})\times\frac{C_{TAP,j,y}}{C_{BASE,j,y}} \times F_{C_{on-road,j,y}}$ | (S16) |
| $\Delta C_{POLICY,j,y}=\Delta C_{C_{POLICY,j,y}}\times\frac{\Delta C_{C_{TOL,j,y}}}{\sum_{POLICY=1}^{5} \Delta C_{C_{POLICY,j,y}}}, POLICY=SEQ,POV,IFE,OTS,PNV$ | (S17) |
| $\Delta C_{PWPOLICY,y}=\frac{\sum_{j} \Delta C_{POLICY,j,y}\times{POP}_{j,y}}{\sum_{j} {POP}_{j,y}}, POLICY=SEQ,POV,IFE,OTS,PNV$ | (S18) |

where $C$ denotes the simulated surface concentrations (that is, PM_2.5_ or summer MDA8 O_3_), with subscripts indicating the scenario (e.g., BASE) or TAP; $F_{C_{on-road,j}}$ represents the fractions of simulated on-road contributions to surface concentrations in grid $j$; $\Delta C_{C}$ is the changes in on-road emissions associated surface concentrations directly calculated from the CAMx simulations; ${POP}_{j}$ represents the total population amount in grid $j$. The gridded population distributions for 2015 and 2020 are obtained from the Global Population for the World (GPW) dataset [37].

**Supplementary Note 5: Simulations of avoided premature deaths attributable to control policies**

Premature deaths associated with exposure to ambient PM_2.5_ and O_3_ are critical metrics for policy assessments [38-40]. The GEMM model is employed to estimate non-accidental mortality associated with non-communicable diseases (NCD) and lower respiratory infections (LRI) due to PM_2.5_ exposure [41]. The GEMM model is developed using PM_2.5_ exposure and premature mortality risk datasets derived from a global range of PM_2.5_ exposure cohort studies, with a particular emphasis on studies conducted in high-pollution regions like China [39, 41]. Currently, the GEMM model is the preferred choice for estimating PM_2.5_-related health burdens in China and other regions [30, 42]. The GEMM NCD + LRI parameterizes the relative risk (RR) of NCD + LRI as a function of PM_2.5_ concentrations ($C_{{PM}_{2.5}}$) as follows:

| $RR(C_{{PM}_{2.5}})=e^{\frac{\theta\times\ln(\frac{z}{\alpha}+1)}{1+e^{(-\frac{z-\mu}{\nu})}}}$, where $z=max(0, C_{{PM}_{2.5}}-2.4)$ | (S19) |
| --- | --- |

where $C_{{PM}_{2.5}}$ represents the annual average PM_2.5_ concentration; $z$ is defined as the maximum of 0 and $C_{{PM}_{2.5}}-2.4$; the shape of PM_2.5_-mortality relationships is determined by the parameters $\theta$, $\alpha$, $\mu$, and $\nu$. According to the GEMM framework, the $RR$ of NCD+LRI associated with PM_2.5_ exposure is calculated by age for adults in 5-year intervals, covering ages from 25 to 85 and beyond. In this study, a distribution of 1,000 point estimations of $\theta$ is calculated using parameters provided by GEMM NCD+LRI and is used to calculate the mean PM_2.5_-attributable premature deaths and the corresponding 95% confidence intervals (CI) [41].

For O_3_ exposure, we adopt the method documented in the GBD study [43], which utilizes data from various cohort studies conducted worldwide to estimate O_3_-related non-accidental mortality attributable to chronic obstructive pulmonary disease (COPD). The GBD study parameterizes the dependence of the relative risk (RR) of COPD on O_3_ concentrations ($C_{O_{3}}$) as:

| $RR(C_{O_{3}})=\left\{ \begin{aligned} e^{\beta\times\left( C_{O_{3}}-C_{0} \right)/10}, &if C_{O_{3}}>C_{0} \\ 1, &otherwise \end{aligned} \right.$ | (S20) |
| --- | --- |

where $C_{O_{3}}$ is the summer MDA8 O_3_; $\beta$ denotes the concentration-response function slope, estimated as 0.061 (95% CI: 0.029-0.093) per 10 ppb of long-term O_3_ exposure, with the theoretical minimum-risk exposure level $C_{0}$ ranging between 29.1 and 35.7 ppb [36]. In this study, 29.1 ppb is used as the minimum-risk exposure level, and a distribution of 1,000 point estimations of $\beta$ is applied to calculate the mean O_3_-attributable premature deaths and the corresponding 95% CI.

The attributable fraction ($AF$) of mortality due to air pollution exposure to component $c$ (i.e., PM_2.5_ and O_3_) in grid $j$ ($AF\left( C_{c,j} \right)$) is further calculated as:

| $AF\left( C_{c,j} \right)=\frac{RR\left( C_{c,j} \right)-1}{RR\left( C_{c,j} \right)}$ | (S21) |
| --- | --- |

The premature mortality ($M$) attributable to PM_2.5_ and O_3_ exposure caused by on-road emissions for a population subgroup $g$ (categorized by age) in grid $j$ ($M_{g}\left( C_{c,j} \right)$) can be further estimated as follows:

| $M_{g}\left( C_{c,j} \right)={POP}_{j}\times{PS}_{g}\times B_{c,g}\times{AF}_{g}\left( C_{c,j} \right)\times F_{C_{on-road,j}}$ | (S22) |
| --- | --- |

where ${PS}_{g}$ is the fraction of a population subgroup $g$ relative to the total population in China; $B_{c,g}$ denotes the national baseline mortality incidence rate of NCD+LRI or COPD for population subgroup $g$; $F_{C_{on-road,j}}$ represents the fraction of simulated on-road contributions to surface concentrations. The national baseline mortality incidence and demographic information are collected from the GBD 2021 study [44]. The total premature deaths in grid $j$ are calculated by summing all population subgroups in this grid.

The total number of avoided premature deaths resulting from the implementation of the five control policies is calculated as the difference between the NoCtrl and BASE scenarios. The impacts of each policy on premature deaths are subsequently estimated using the direct proportion approach [45], which assumes that the proportion of avoided premature deaths related to PM_2.5_ or O_3_ attributed to each policy is directly proportional to its contribution to the total change in PM_2.5_ or O_3_ concentrations. This direct proportion approach has been validated by the GBD MAPS study [45] and has been widely adopted by other studies [30, 46, 47]. The total changes in premature mortality ($\Delta M$) due to the five policies in year $y$ can be expressed as:

| $\Delta M_{j,y}=M_{NoCtrl,j,y}-M_{BASE,j,y}=\sum_{POLICY=1}^{5} \Delta M_{POLICY,j,y}=\sum_{POLICY=1}^{5} \Delta M_{j,y}\times\frac{\Delta C_{POLICY,j,y}}{\Delta C_{C_{TOL,j,y}}},POLICY=SEQ,POV,IFE,OTS,PNV$ | (S23) |
| --- | --- |

where $\Delta M_{POLICY}$ represents the policy-specific mortality changes attributed to air pollution exposure caused by on-road emissions; $M_{NoCtrl}$and $M_{BASE}$ denote the premature deaths related to on-road transportation emissions under the NoCtrl and BASE scenarios, respectively. The national-level health impacts are calculated by summing the avoided deaths across all grid cells in China.

**Supplementary Note 6: Development of the Synergy Index**

The Synergy Index (SynI) is developed to quantify the synergistic effects of environmental policies on both protecting public health and reducing GHG emissions, and the difference in their impacts between the health and climate dimensions. Traditional approaches to policy evaluation typically assess health impacts and GHG emission reductions separately. The Synergy Index aims to provide a more integrated evaluation by capturing the combined effects of policies that influence both health and climate, reflecting the extent to which environmental policies align with national carbon-pollution co-control goals. The use of the Coupling Coordination Degree (CCD) model serves as the foundation for this index, as it is an established method for quantifying the degree of interaction between multiple systems, including carbon and air pollutant emissions [48-50]. In this case, the two systems of interest are health benefits, measured in terms of avoided premature deaths, and climate benefits, measured in terms of reductions in CO_2_e emissions. The structure of the formula is intended to simultaneously account for the interaction between health and climate metrics as well as the relative importance of each in the overall policy evaluation. The Synergy Index is mathematically expressed as:

| $SynI=\sqrt{\frac{2\sqrt{HM\times CM}}{HM+CM}\times(\alpha HM+\beta CM)}$ | (S24) |
| --- | --- |

where $HM$ and $CM$ represent the health and climate metrics, respectively; $\alpha$ and $\beta$ are the weights of subsystems, with $\alpha+\beta=1$. Due to the equal importance of air quality-associated health and climate, $\alpha$ and $\beta$ are set to 0.5. The values of $HM$, $CM$, and $SynI$ range from 0 to 1.

The geometric mean in the first term of the formula introduces non-linearity, increasing the sensitivity of the index to imbalances between the health and climate dimensions. Specifically, the first component, $\frac{2\sqrt{HM\times CM}}{HM+CM}$ attains a value of 1 only when HM and CM are equal and declines as their values diverge. This behavior arises because the geometric mean $\sqrt{HM\times CM}$ increases more slowly than the arithmetic mean $HM+CM$ when one dimension becomes small. It ensures that when health and climate effects are uncoordinated or contradictory—i.e., one improves while the other worsens—the synergy index decreases, preventing an overestimation of the policy’s overall benefits. Conversely, when both metrics improve in parallel, the geometric mean amplifies this effect, reflecting a higher degree of synergy. The second term in the formula introduces weights for the health and climate metrics, representing their relative importance in the overall synergy evaluation. In the context of carbon-pollution co-control, equal weights ($\alpha= \beta= 0.5$) are assumed, signifying the equal importance of air quality-associated health and climate benefits. Both health and climate metrics are normalized based on relative changes to a common baseline, such as the contributions of on-road transportation in 2015. This normalization process converts the metrics into dimensionless quantities, enabling consistent impact comparisons across varying policies and periods. Finally, the square root in the formula prevents one dimension from being disproportionately emphasized, particularly when one metric improves significantly while the other does not. It also smooths the interaction between the two dimensions, ensuring that the index reflects synergy when both health and climate metrics improve together and reduces the index when the outcomes are misaligned. In summary, the Synergy Index provides a robust framework for evaluating the degree of health and climate benefits attributable to environmental policies, capturing the intricacies of their interdependence. By integrating these two dimensions into a single, unified measure, the index facilitates policy assessment in a way that accounts for both their individual and joint effects, offering valuable insights into overall effectiveness.

**Supplementary Note 7: Design of enhanced structural adjustment scenarios for 2020**

This study evaluates the potential synergies resulting from structural transitions in China’s on-road transportation sector, with a focus on optimizing the vehicle fleet and transportation structure to meet the 2025 targets. Three counterfactual scenarios are designed: *Strengthen Transportation Structure Transition (TraTran)*, *Strengthen Vehicle Fleet Structure Transition (FleTran)*, and *Combined Structure Transition (StrTran)*. To isolate the effects of these structural changes, all other socioeconomic and policy parameters are held constant at their 2020 levels. Each scenario assumes that, by 2020, China had already achieved the vehicle and transportation structural transition targets proposed in the Fourteenth Five-Year Plan of the People’s Republic of China (14^th^ FYP; 2021-2025) [51]. These targets include the accelerated phase-out of outdated vehicles, a rising penetration rate of EVs in new vehicle sales, and an increased fractions of alternative transportation modes beyond on-road transportation. The key assumption parameters are originally compiled based on China’s official 14^th^ FYP. A detailed description of these scenarios is provided below.

**Strengthen Transportation Structure Transition (TraTran).** As outlined in the 14^th^ FYP, optimizing passenger and freight transportation structures is a key priority. The complementary “Work Plan for Promoting the Development of Multimodal Transportation and Optimizing and Adjusting the Transportation Structure (2021-2025)” further specifies that by 2025, the fractions of rail freight are expected to increase by 10% compared to 2020, and the fractions of water freight are projected to grow by 12% over the same period [52]. In the “TraTran” scenario in 2020, we assume a reduction in the fractions of on-road passenger and freight turnover compared to the 2020 baseline, reflected in $\eta^{'}$ in Eq. (S1). For the passenger subsector, which lacks explicit targets in the plan, the reduction in the fractions of on-road passenger turnover between 2020 and 2025 is assumed to follow the trend observed from 2015 to 2020. For freight transport, we incorporate the 2025 targets by assuming a 10% increase in the fractions of rail freight and a 12% increase in the fractions of water freight, in line with the targets specified in the plan.

**Strengthen Vehicle Fleet Structure Transition (FleTran)**. According to the 14^th^ FYP, by 2025, all provinces are required to accelerate the phase-out of China 3/Ⅲ vehicles. The plan also establishes specific targets for EV penetration, stating that EVs should account for 20% of new vehicle sales, while public transportation vehicles, including buses and taxis, should achieve an 80% penetration rate. Based on these targets, we assume that in the “FleTran” scenario in 2020, the total vehicle population and the number of newly registered vehicles remain unchanged. However, both the emission standards and fuel composition of these vehicles undergo modifications, as reflected in ${{VP}^{'}}_{s,f}$ in Eq. (S1). Specifically, the analysis assumes that the phase-out of China 3/Ⅲ vehicles is initially offset by an equivalent number of newly purchased vehicles. Subsequently, while maintaining a constant total number of newly registered vehicles in 2020, a designated proportion of these vehicles must be electric to align with the mandated EV penetration targets. The remaining portion of the fleet consists of internal combustion engine vehicles (ICEVs) that comply with the China 6/Ⅵ emission standards. The EV penetration rates are defined based on both official policies and recent government research [51, 53, 54]. For passenger vehicles—including light-duty, medium-duty, and heavy-duty categories—EVs are expected to constitute 20% of newly registered vehicles. Public transportation vehicles, such as buses and taxis, are subject to stricter electrification targets, with an 80% penetration rate mandated by the plan. In contrast, EV adoption for freight transport is expected to be more gradual, with light-duty trucks, medium-duty trucks, and heavy-duty trucks reaching penetration rates of 10%, 6%, and 6%, respectively.

**Combined Structure Transition (StrTran).** This scenario combines the assumptions from *Strengthen Transportation Structure Transition* and *Strengthen Vehicle Fleet Structure Transition*, meaning that all structural adjustment targets related to on-road vehicles mentioned in the 14^th^ FYP have been achieved. These adjustments are reflected in ${{VP}^{'}}_{s,f}$ and $\eta^{'}$ in Eq. (S1) simultaneously.

The estimation of air pollution and GHG emission reductions, air quality improvements, avoided deaths, and the Synergy Index attributable to each scenario follows the methods documented in Supplementary Notes 2-6, with reductions calculated as emissions in the BASE case minus those in the corresponding counterfactual scenario. The air quality and health impacts of each structural transition design are assessed by calculating the differences in PM_2.5_ and O_3_ exposure, as well as the corresponding premature deaths attributable to on-road emissions, between the Counterfactual and BASE scenarios for the respective year.

**Supplementary Note 8: Uncertainties and limitations**

The estimated synergies of on-road transportation emission control in China are primarily influenced by multiple sources of uncertainty in the assessment of climate and health impacts. Supplementary Fig. 1-2 and Fig. 4 illustrate the corresponding uncertainty ranges, with detailed discussions on each step provided below.

First, the estimated CO_2_e emission reductions are subject to uncertainty due to both the CO_2_ emission inventories and the choice of the GWP_20_ value for BC First, the estimated CO_2_e emission reductions are subject to uncertainty due to both the CO_2_ emission inventories and the choice of the GWP_20_ value for BC. The baseline GHG emission inventories for on-road transportation in our study were compared with those reported in previous studies [55-59], which are well recognized for their comprehensive assessments of long-term GHG inventories in Chinese transportation. Our estimated emission trends closely align with the findings reported in the aforementioned studies [4]. In addition, we adopted the GWP_20_ value for BC as estimated by Tanaka et al. [60] to account for uncertainties in CO_2_e emissions, which was estimated GWP_20_ value for BC as 1,189 ± 854.39. This GWP_20_ value for BC was derived from extensive multi-model simulations, with its underlying methodologies and assumptions having been rigorously validated in previous climate impact assessments, thereby ensuring its robustness [61-65]. The uncertainties of CO_2_e are represented in Supplementary Fig. 2, with error bars denoting ±1σ around the mean.

Second, the PM_2.5_ and O_3_ concentrations simulated by the WRF-CAMx modeling system are inevitably influenced by inherent uncertainties in the input emission inventories and the representation of chemical and physical processes within the model. To mitigate these uncertainties, the baseline on-road transportation emission inventories used in this study were compared with those from previous studies [9, 55-57, 66, 67], and the resulting emission trends showed good agreement with earlier reports [4]. For all other anthropogenic emissions, we used data from the MEIC model, a widely applied and well-validated tool for air quality simulations, as confirmed by comparisons with both surface and satellite-based observations [26, 30, 68]. Uncertainties in the simulations were quantified by comparing model-derived PM_2.5_ and O_3_ concentrations against ground-based and machine-learning-base data, with an emphasis on population-weighted metrics to better assess health impacts. Specifically, errors in the WRF-CAMx simulations are expressed as the normalized mean bias (NMB) between CAMx and machine learning-based concentration estimates. Over the two years, the national NMB for population-weighted PM_2.5_ ranges from -0.3% to 6.6%, and for population-weighted summer MDA O_3_, it ranges from -0.1% to 13.3% (statistics in red texts in Supplementary Fig. 4).

Third, our estimates of premature mortality are subject to uncertainty due to the limited epidemiological evidence underlying the exposure-response relationships. For PM_2.5_-related mortality, we quantify uncertainties in the GEMM functions using Monte Carlo simulations, which generate 1,000 estimates of $\theta$ based on parameters provided by the GEMM NCD+LRI model, as described in Burnett et al.[41]. For O_3_-related mortality, uncertainty is represented by the 95% confidence intervals for the relative risk of respiratory COPD mortality associated with long-term O_3_ exposure, estimated at 1.06 (95% CI: 1.02-1.10) per 10 ppb increase, as reported in GDB 2019 [43]. Uncertainties related to population and baseline mortality were not incorporated into our estimates, as supported by previous studies indicating that these factors contribute only minimally to the confidence intervals for premature mortality [30, 69]. Given these, we conclude that excluding uncertainties introduced by population and baseline mortality does not affect our conclusions.

Fourth, the normalization baseline used in calculating the Synergy Index introduces an additional source of methodological uncertainty. To ensure comparability of health and climate metrics across policies and periods, the Synergy Index normalizes both components against 2015 totals of CO_2_e emissions and premature deaths. However, to evaluate whether the choice of baseline year affects values for policies whose main impacts occur in later periods, we conducted a sensitivity analysis using 2020 totals as an alternative baseline (Supplementary Table 13). The results show that while some values in Period II exhibit slight upward or downward variations, the overall synergy patterns and policy rankings remain consistent, confirming that our conclusions are robust to the choice of normalization year.

Moreover, the alternative health-climate weightings may also influence the Synergy Index, we conducted a sensitivity analysis by varying the health weight from 0.1 to 1.9 in increments of 0.1, while adjusting the climate weight so that the two always sum to unity. The results (Supplementary Fig. 5) show policy-specific Synergy Index differs across weighting choices, but the main conclusions of our study remain robust. Across all weightings, the downward trend in the Synergy Index from 2015 to 2020 is sustained, the synergies associated with structural transition scenarios remain enhanced, and the *Combined Structure Transition* scenario continues to demonstrate the greatest overall performance. Furthermore, when the weighting shifts toward the health dimension, policies such as *Strengthen Standard and Fuel Quality*, *Phase Out Outdated Vehicle*, and *Strengthen Vehicle Fleet Structure Transition* exhibit higher Synergy Index scores. In contrast, policies with relatively stronger climate impacts, including *Improve Fuel Efficiency* and *Strengthen Transportation Structure Transition*, achieve better performance under scenarios that emphasize the climate component. The *Promote New Energy Vehicle* policy shows little sensitivity to weighting adjustments, since its contributions to both health and climate outcomes remain limited.

*Supplementary Figures:*


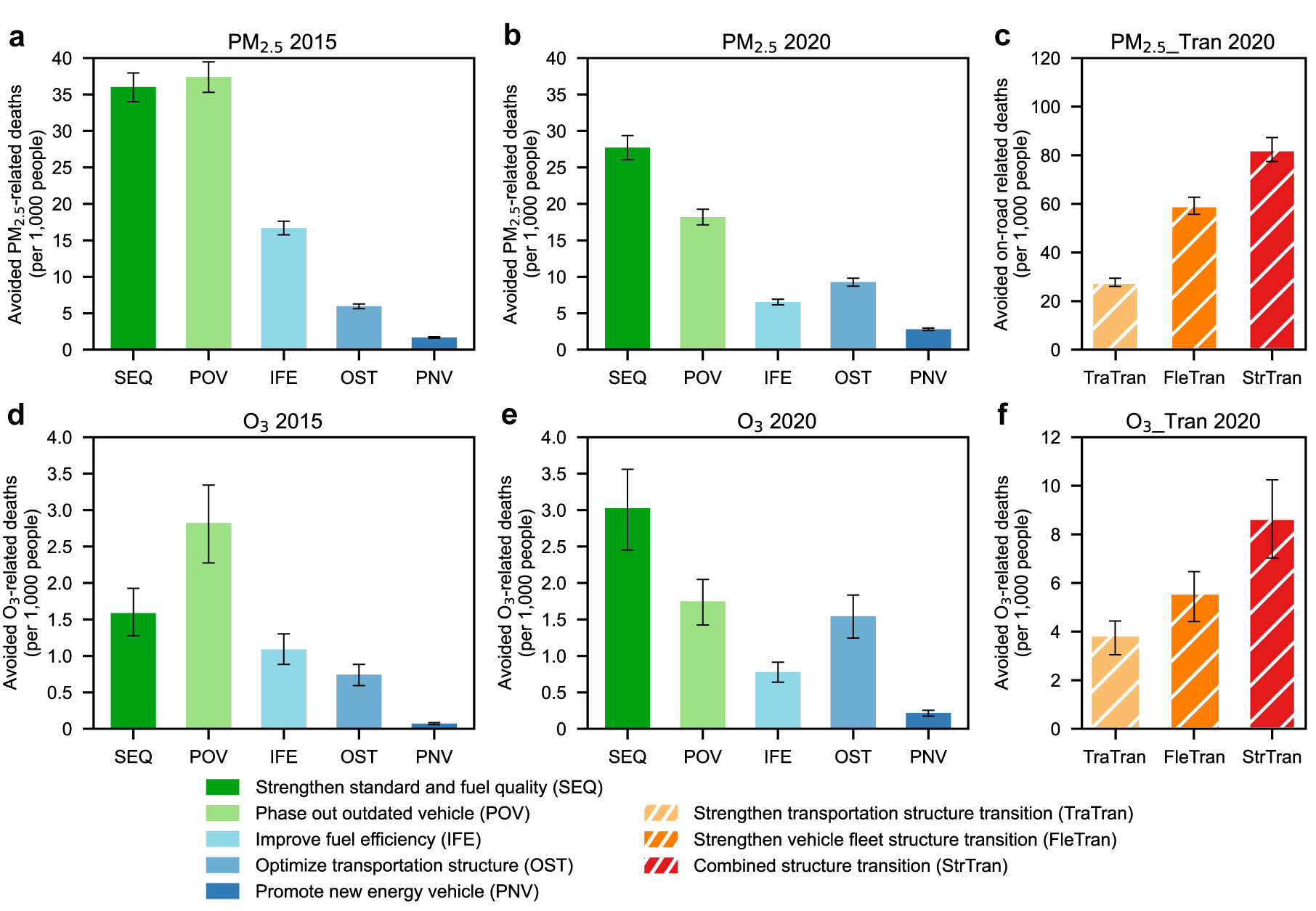


**Supplementary Figure 1 | Estimated avoided premature deaths contributed by different policies during two periods. (a-b**) Avoided PM_2.5_-related deaths associated with five emission control policies in 2015 **(a)**, and in 2020 **(b)**. (**c**) Avoided PM_2.5_-related deaths associated with enhanced structural adjustment in 2020. (**d-e**) Avoided O_3_-related deaths associated with five emission control policies in 2015 **(d)** and in 2020 **(e)**. (**f**) Avoided O_3_-related deaths associated with enhanced structural adjustment in 2020. Error bars show the 95% CI of our estimates.


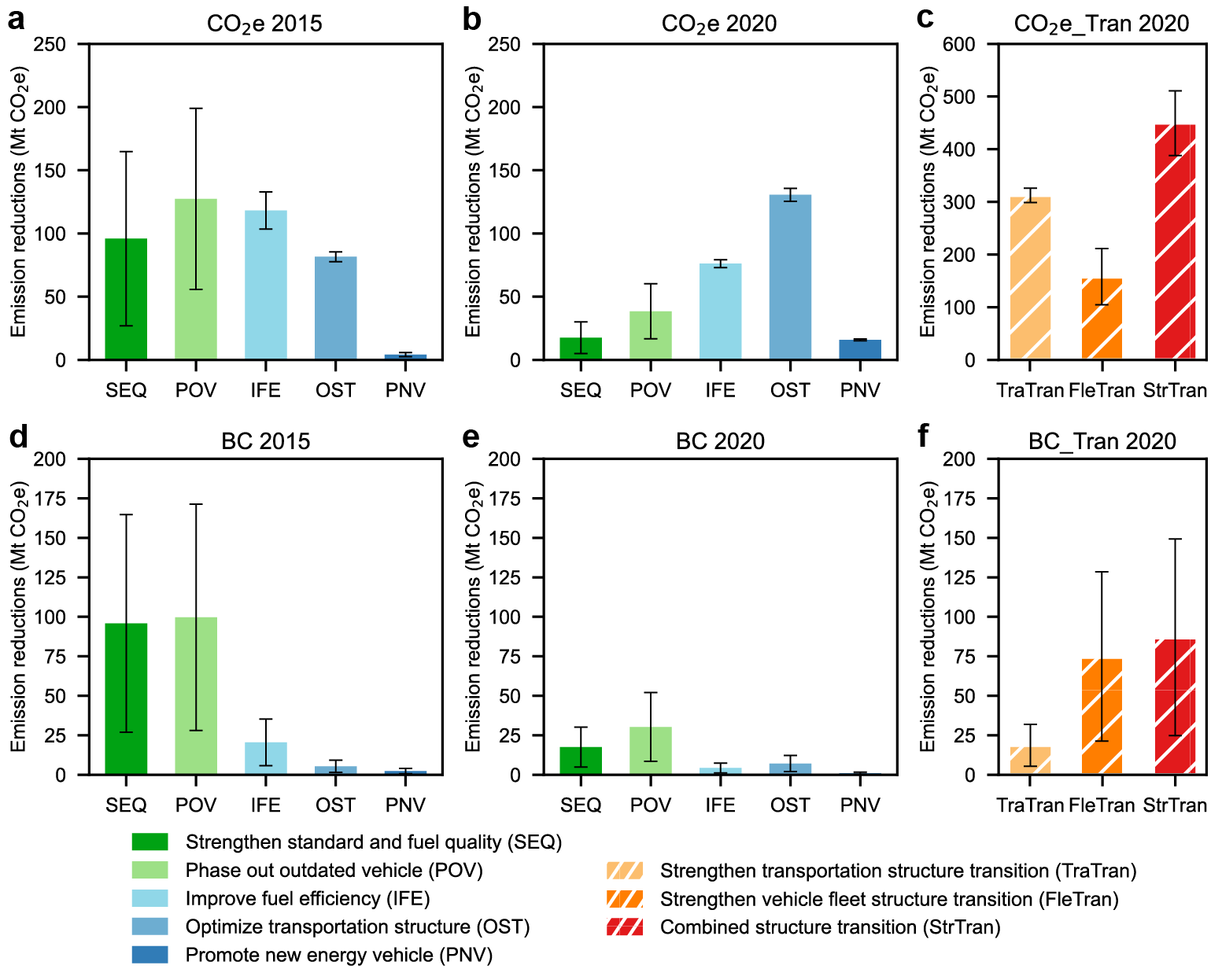


**Supplementary Figure 2 | Estimated GHG emission reductions contributed by different policies during two periods. (a-b**) Estimated GHG emission reductions associated with five emission control policies in 2015 **(a)** and in 2020 **(b)**. **(c**) Estimated in GHG emission reductions in scenarios with enhanced structural adjustment in 2020. (**d-e**) Estimated BC emission reductions based on CO_2_e associated with five emission control policies in 2015 **(d)** and in 2020 **(e)**. (**f**) Estimated BC emission reductions based on CO_2_e in scenarios with enhanced structural adjustment in 2020. Error bars indicate uncertainty arising from metrics as ± 1σ around the mean as reported in recent studies [60, 65]. Changes in CO_2_ and BC are considered.

**
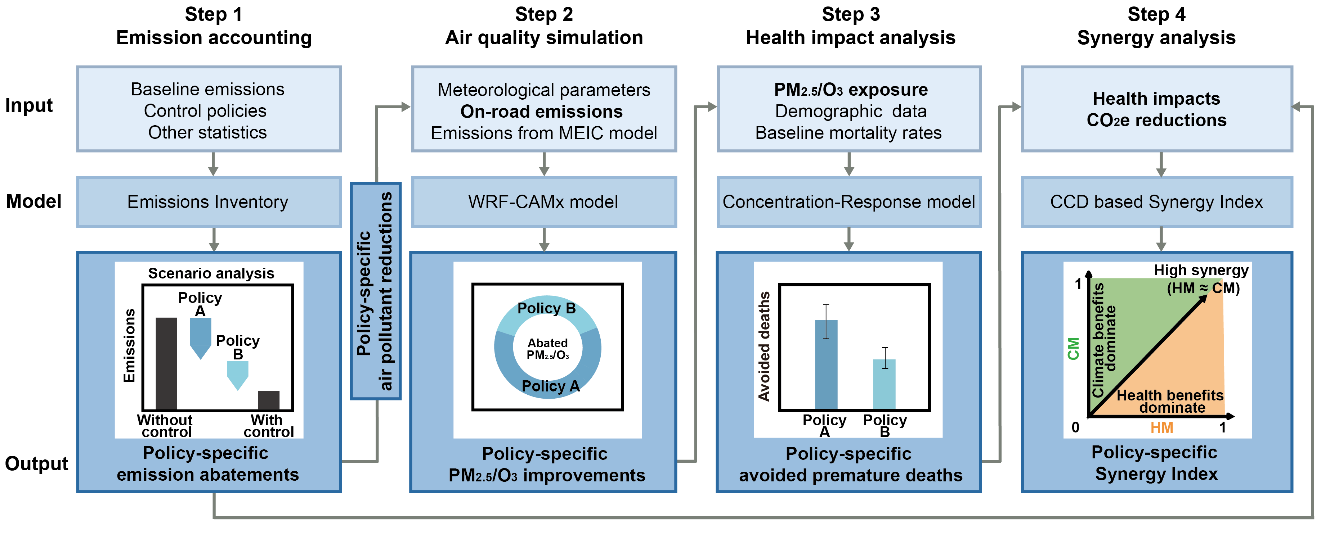
**

**Supplementary Figure 3 | Methodology framework to evaluate the synergetic impacts of emission control policies in China’s on-road transportation sector. The MEIC and CCD model represent the Multi-resolution Emission Inventory for China and Coupling Coordination Degree model, respectively.**


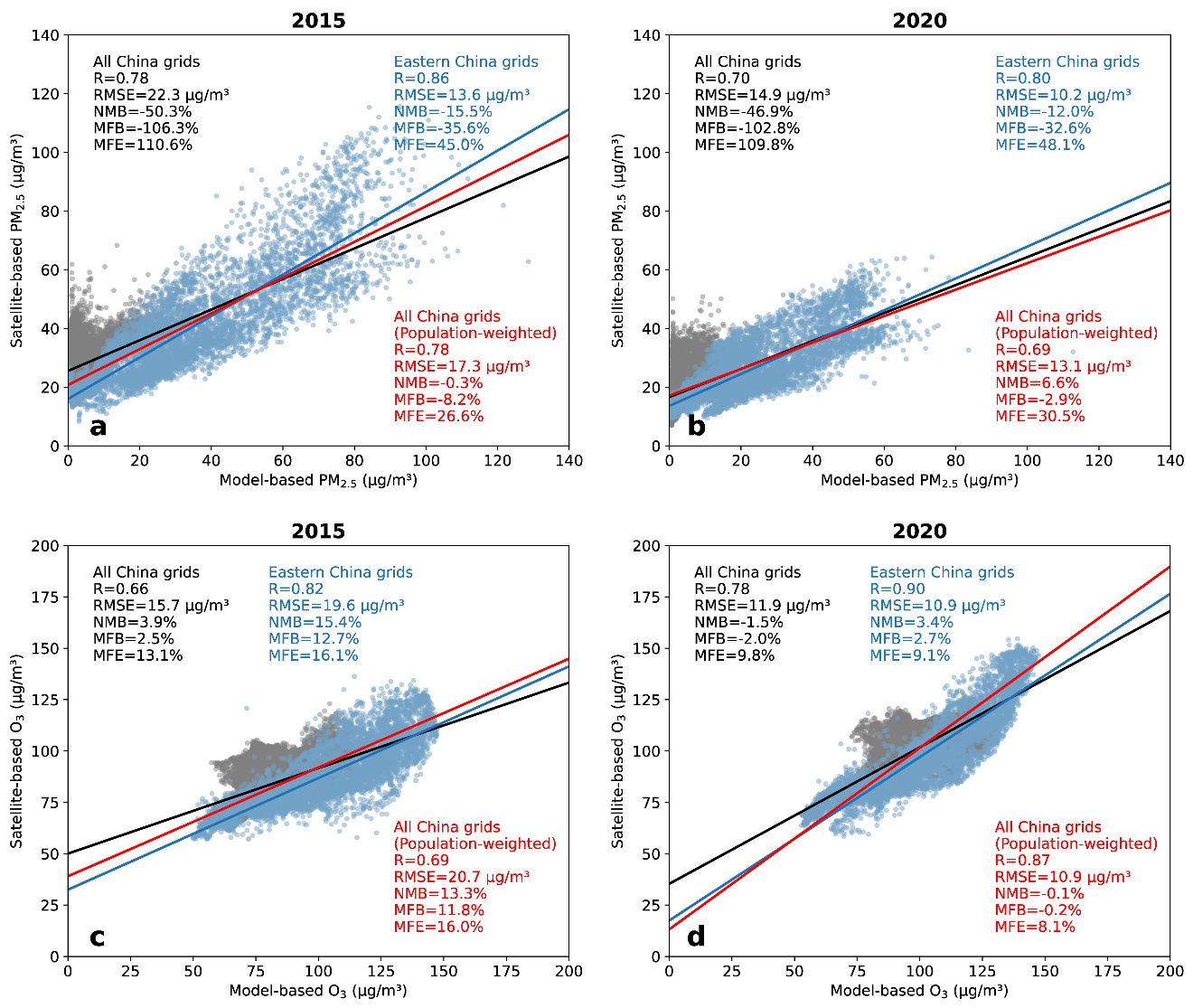


**Supplementary Figure 4 | Comparison between simulated and machine-learning-based PM_2.5_ and O_3_ concentrations collected from Tracking Air Pollution in China dataset. (a-b**) Results for annual mean PM_2.5_ concentrations in 2015 (**a**) and 2020 (**b**). (**c-d**) Results for warm season mean MDA O_3_ concentrations in 2015 (**c**) and 2020 (**d**). Blue dots depict grids located in the eastern part of China and the grey dots depict grids located in the western part of China. The blue line and blue texts show results for grids located in the eastern part of China; the black line and black texts in each panel show the regression and evaluation results for all grids; the red line and red texts show population-weighed regression and evaluation results for all grids. The definitions of the eastern and western parts of China are presented in Supplementary Table 8, which are consistent with Geng et al. [30].

**
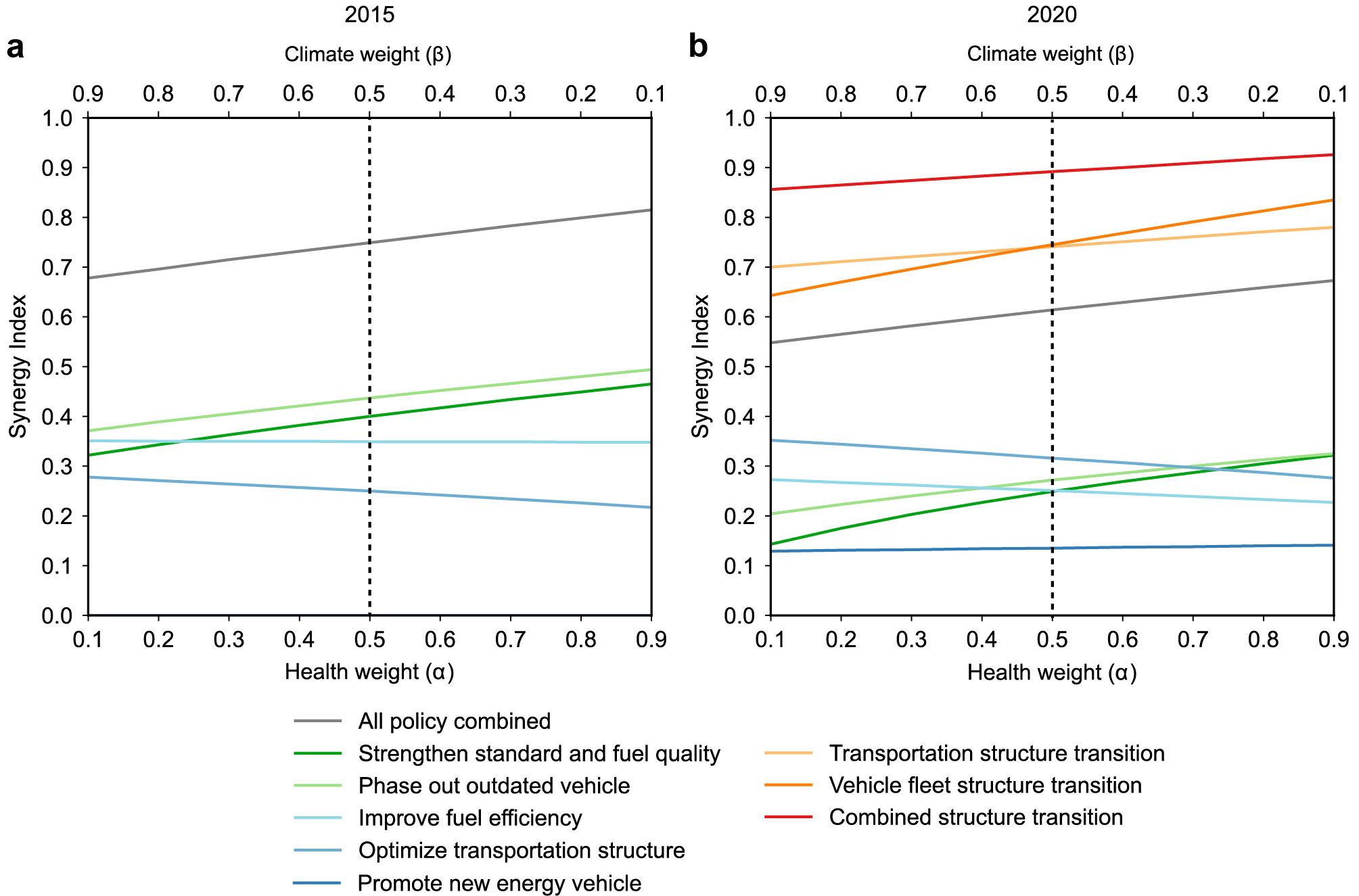
**

**Supplementary Figure 5 | Sensitivity of Synergy Index to health climate weightings.**

*Supplementary Tables:*

Supplementary Table 1 | Vehicle classification in this study.

| Vehicle type | Fuel type | Standard^*^ | |
| --- | --- | --- | --- |
| Light-duty passenger vehicle (LDPV) | Gasoline, alternative-energy | China 0-6 | |
| Medium-duty passenger vehicle (MDPV) | Gasoline, diesel, alternative-energy | China 0-6/Ⅵ | |
| Heavy-duty passenger vehicle (HDPV) | Gasoline, diesel, alternative-energy | China 0-6/Ⅵ | |
| Bus | Gasoline, diesel, alternative-energy | China 0-6/Ⅵ | |
| Taxi | Gasoline, alternative-energy | China 0-6 | |
| Light-duty truck (LDT) | Gasoline, diesel, alternative-energy | China 0-6/Ⅵ | |
| Medium-duty truck (MDT) | Diesel | China 0-Ⅵ | |
| Heavy-duty truck (HDT) | Diesel | China 0-Ⅵ | |
| Motorcycle (MC) | Gasoline | China 0-3 | |
| ^*^Note: the emission standards for gasoline vehicles are written with Arabic numerals [1–6], while the standards for diesel vehicles are indicated using Roman numerals [Ι-Ⅵ]. | | |  |

Supplementary Table 2 | Timeline of the implementation of emission standards after 2010.

| Vehicle type | Region | China 4/Ⅳ | China 5/V | China 6/Ⅵ |
| --- | --- | --- | --- | --- |
|  |  |  |  |  |
| China- HDPV-G | Nation | 2013 | 2017 | 2020 |
|  | Beijing | - | 2013 | 2020 |
|  | Shanghai | - | 2014 | 2020 |
| China-MDPV-G | Nation | 2013 | 2017 | 2020 |
|  | Beijing | - | 2013 | 2020 |
|  | Shanghai | - | 2014 | 2020 |
| China-LDPV-G | Nation | 2011 | 2017 | 2020 |
|  | Beijing | - | 2013 | 2020 |
|  | Shanghai | - | 2014 | 2020 |
| China-LDT-G | Nation | 2011 | 2017 | 2020 |
|  | Beijing | - | 2013 | 2020 |
|  | Shanghai | - | 2014 | 2020 |
| China-HDPV-D | Nation | 2013 | 2017 | 2020 |
|  | Beijing | - | 2013 | 2020 |
|  | Shanghai | - | 2014 | 2020 |
| China-MDPV-D | Nation | 2013 | 2017 | 2020 |
|  | Beijing | - | 2013 | 2020 |
|  | Shanghai | - | 2014 | 2020 |
| China-HDT-D | Nation | 2013 | 2017 | 2020 |
|  | Beijing | - | 2013 | 2020 |
|  | Shanghai | - | 2014 | 2020 |
| China-MDT-D | Nation | 2013 | 2017 | 2020 |
|  | Beijing | - | 2013 | 2020 |
|  | Shanghai | - | 2014 | 2020 |
| China-LDT-D | Nation | 2013 | 2017 | 2020 |
|  | Beijing | - | 2013 | 2020 |
|  | Shanghai | - | 2014 | 2020 |

Supplementary Table 3 | Summary of air quality improvement, avoided premature deaths, and GHG emission reductions associated with five emission control policies in 2015 and in 2020.

| Policy | Year^(1)^ | Air quality improvement | | Avoided premature deaths associated with exposure | | GHG emission reductions (CO_2_e ) | | |
| --- | --- | --- | --- | --- | --- | --- | --- | --- |
|  |  | PW PM_2.5_^(2)^ (μg/m^3^) | PW MDA8 O_3_^(3)^ (μg/m^3^) | PM_2.5_  exposure  (95% CI) | O_3_  exposure  (95% CI) | CO_2_  (Mt) | BC  (Mt) | Total CO_2_e  (Mt) |
| Strengthen Standard and Fuel Quality  (SEQ) | 2015 | 1.03 | 1.35 | 36,021  (34,018, 37,967) | 1,587  (1,277, 1,926) | 0 | 95.85  (26.97, 164.72) | 95.85  (26.97, 164.72) |
|  | 2020 | 0.60 | 2.02 | 27,716  (26,054, 29,371) | 3,026  (2,451, 3,560) | 0 | 17.52  (4.93, 30.11) | 17.52  (4.93, 30.11) |
| Phase Out Outdated Vehicle  (POV) | 2015 | 1.07 | 2.33 | 37,404  (35,298, 39,465) | 2,823  (2,276, 3,343) | 27.67 | 99.66  (28.05, 171.28) | 127.33  (55.72, 198.95) |
|  | 2020 | 0.39 | 1.16 | 18,192  (17,118, 19,266) | 1,749  (1,424, 2,048) | 8.15 | 30.28  (8.52, 52.04) | 38.43  (16.67, 60.18) |
| Improve Fuel Efficiency (IFE) | 2015 | 0.47 | 0.93 | 16,683  (15,746, 17,613) | 1,088  (884, 1,302) | 97.61 | 20.52  (5.78, 35.27) | 118.14  (103.39, 132.89) |
|  | 2020 | 0.14 | 0.51 | 6,532  (6,146, 6,924) | 778  (640, 914) | 71.83 | 4.33  (1.22, 7.44) | 76.16  (73.05, 79.27) |
| Note: (1) The benefits reported for 2015 and 2020 represent the cumulative impacts of policy implementation up to the final year of Period I and Period II, respectively; (2) The PW PM_2.5_ represents the national population-weighted annual mean PM_2.5;_ (3) The PW MDA8 O_3_ represents the national population-weighted summer maximum daily 8-hour mean O_3_. | | | | | | | | |

Supplementary Table 3 | Summary of air quality improvement, avoided premature deaths, and GHG emission reductions associated with five emission control policies in 2015 and in 2020 (continued).

| Policy | Year^(1)^ | Air quality improvement | | Avoided premature deaths associated with exposure | | GHG emission reductions (CO_2_e ) | | |
| --- | --- | --- | --- | --- | --- | --- | --- | --- |
|  |  | PW PM_2.5_^(2)^ (μg/m^3^) | PW MDA8 O_3_^(3)^ (μg/m^3^) | PM_2.5_  exposure  (95% CI) | O_3_  exposure  (95% CI) | CO_2_  (Mt) | BC  (Mt) | Total CO_2_e  (Mt) |
| Optimize Transporta-tion Structure (OTS) | 2015 | 0.18 | 0.59 | 5,951  (5,621, 6,283) | 742  (593, 884) | 76.19 | 5.38  (1.51, 9.24) | 81.57  (77.70, 85.43) |
|  | 2020 | 0.19 | 1.07 | 9,264  (8,707, 9,807) | 1,544  (1,245, 1,835) | 123.45 | 7.11  (2.00, 12.21) | 130.56  (125.45, 135.66) |
| Promote New Energy Vehicle (PNV) | 2015 | 0.05 | 0.06 | 1,668  (1,575, 1,759) | 71  (57, 84) | 1.80 | 2.35  (0.66, 4.04) | 4.15  (2.46, 5.84) |
|  | 2020 | 0.06 | 0.15 | 2,799  (2,633, 2,965) | 217  (175, 255) | 14.78 | 0.96  (0.27, 1.65) | 15.74  (15.05, 16.43) |
| Total | 2015 | 2.80 | 5.26 | 97,727  (92,258, 103,087) | 6,311  (5,087, 7,539) | 203.28 | 223.76  (62.97, 384.55) | 427.04  (266.24, 587.83) |
|  | 2020 | 1.39 | 4.92 | 64,503  (60,659, 68,333) | 7,314  (5,935, 8,612) | 218.21 | 60.2  (16.94, 103.45) | 278.41  (235.15, 321.65) |
| Note: (1) The benefits reported for 2015 and 2020 represent the cumulative impacts of policy implementation up to the final year of Period I and Period II, respectively; (2) The PW PM_2.5_ represents the national population-weighted annual mean PM_2.5;_ (3) The PW MDA8 O_3_ represents the national population-weighted summer maximum daily 8-hour mean O_3_. | | | | | | | | |

Supplementary Table 4 | Design of Policy-specific scenarios.

| Scenario | Assumption | Parameters modified in Eq. (S1) | Data source |
| --- | --- | --- | --- |
| Strengthen Standard and Fuel Quality (SEQ) | New vehicles remain at base-year emission factor levels; fuel quality fixed at base-year levels | Emission factors ($EF'$) | Timeline of the implementation of emission standards after 2010 in Supplementary Table 2 [4] and the Technical Guidelines on Emission Inventory in China [7] |
| Phase Out  Outdated Vehicle (POV) | No additional phase-out actions take place after the base year; overall vehicle stock held constant | Vehicle population ($VP'$) | The China Mobile Source Environmental Management Annual Report [9], Zhou et al. [15], Zhang et al. [2], and Geng et al. [3]. |
| Improve Fuel Efficiency (IFE) | No further improvements in fuel efficiency after the base year consumption | Emission factor adjustment ($EF'$) and fuel efficiency ($FE$) | The Fuel Consumption Limits for Heavy-Duty Commercial Vehicles [70], Fuel Consumption Standards for Passenger Vehicles [5], the Fuel Economy and Environmental Impacts (FEEI) model [19] and Liu et al. [67] |
| Optimize Transportation Structure (OTS) | Shares of on-road passenger and freight turnover fixed at base-year levels | On-road transportation demand factor ($\eta'$) | Transportation Development Statistics Bulletin [6] |
| Promote New Energy Vehicle (PNV) | No further NEV deployment beyond the base year; new vehicle registrations held constant | Vehicle population (${{VP}^{'}}_{f}$) | Global EV Outlook [71] and the Chinese Academy of Environmental Planning |

Note: The detailed changes in vehicle population, emission standards, fuel consumption targets, transportation structure, and new energy vehicle deployment during 2010-2020 are presented in Figure 1.

Supplementary Table 5 | List of scenario groups.

| Cases | Number of  Subcases | Year of  meteorology | On-road transportation  emissions | | Other anthropogenic emissions | Notes |
| --- | --- | --- | --- | --- | --- | --- |
| *POLICY* | 5 | 2015 | Baseline emissions for 2015 plus the emission reductions from each of the five emission control policies | Strengthen Standard and Fuel Quality (SEQ) | Baseline emissions  for 2015 | To quantify the policy-specific impacts of each emission control policy |
|  |  |  |  | Phase Out Outdated Vehicle (POV) |  |  |
|  |  |  |  | Improve Fuel Efficiency (IFE) |  |  |
|  |  |  |  | Optimize Transportation Structure (OTS) |  |  |
|  |  |  |  | Promote New Energy Vehicle (PNV) |  |  |
|  | 5 | 2020 | Baseline emissions for 2020 plus the emission reductions from each of the five emission control policies | Strengthen Standard and Fuel Quality (SEQ) | Baseline emissions  for 2020 |  |
|  |  |  |  | Phase Out Outdated Vehicle (POV) |  |  |
|  |  |  |  | Improve Fuel Efficiency (IFE) |  |  |
|  |  |  |  | Optimize Transportation Structure (OTS) |  |  |
|  |  |  |  | Promote New Energy Vehicle (PNV) |  |  |
| *Counterfa-ctual* | 3 | 2020 | Baseline emissions for 2020 minus the emission reductions from each of the three structure transition scenarios | Strengthen Transportation Structure Transition (TraTran) | Baseline emissions  for 2020 | To quantify the impacts of each structure transition scenario |
|  |  |  |  | Strengthen Vehicle Fleet Structure Transition (FleTran) |  |  |
|  |  |  |  | Combined Structure Transition (StrTran) |  |  |
| *BASE* | 1 | 2015 | Baseline emissions for 2015 | | Baseline emissions  for 2015 | Baseline scenario for estimating actual conditions under current policy status |
|  | 1 | 2020 | Baseline emissions for 2020 | | Baseline emissions  for 2020 |  |
| *NoCtrl* | 1 | 2015 | Baseline emissions for 2015 plus the total emission reductions from the five emission control policies | | Baseline emissions  for 2015 | No control for normalization |
|  | 1 | 2020 | Baseline emissions for 2020 plus the total emission reductions from the five emission control policies | | Baseline emissions  for 2020 |  |

Supplementary Table 6 | The design features of counterfactual scenarios.

| Scenario | Parameters modified in Eq. (S1) | Parameters held constant in Eq. (S1) | Key assumptions based on 14^th^ FYP | |
| --- | --- | --- | --- | --- |
| Strengthen Transportation Structure Transition  (TraTran) | On-road transportation demand ($\eta^{'}$) | Vehicle population (${VP}^{'}$) and emission factors (${EF}^{'}$) | Fractions of passenger | Trends observed from 2015 to 2020 |
|  |  |  | Fractions of rail freight | a 10% increase |
|  |  |  | Fractions of water freight | a 12% increase |
| Strengthen Vehicle Fleet Structure Transition  (FleTran) | Emission standards and fuel composition of vehicles ${{VP}^{'}}_{s,f}$ | Total vehicle population and the total number of newly registered vehicles (${{VP}^{'}}_{t}$)，emission factors (${EF}^{'}$), and the on-road transportation demand ($\eta^{'}$) | Outdated vehicles | 100% phase-out of China Three vehicles |
|  |  |  | Newly sale vehicles | 20 % EV share of new passenger vehicle sales |
|  |  |  |  | 80% EV share of new public buses and taxis |
|  |  |  |  | 10% EV share of new light-duty trucks |
|  |  |  |  | 6% EV share of new medium- and heavy-duty trucks |
| Combined Structure Transition  (StrTran) | ${{VP}^{'}}_{s,f}$ and $\eta^{'}$ | Total vehicle population and the total number of newly registered vehicles (${{VP}^{'}}_{t}$), and emission factors (${EF}^{'}$) | All adjustments from TraTran and FleTran achieved simultaneously | |

Supplementary Table 7 | Evaluation of meteorological parameters simulated by the WRF model.

| Variable | Year | Sample number | R | Mean Observation | Mean Simulation | MB | GE | RMSE | NMB (%) | NME (%) |
| --- | --- | --- | --- | --- | --- | --- | --- | --- | --- | --- |
| Temperature (°C) | 2015 | 4493251 | 0.97 | 13.97 | 13.52 | -0.45 | 2.40 | 3.26 | -3.22 | 17.16 |
|  | 2020 | 5115638 | 0.97 | 14.48 | 13.94 | -0.54 | 2.41 | 3.27 | -3.70 | 16.64 |
| Relative humidity  (%) | 2015 | 4481958 | 0.75 | 68.59 | 67.34 | -1.25 | 11.78 | 15.82 | -1.82 | 17.18 |
|  | 2020 | 5104166 | 0.76 | 68.65 | 67.07 | -1.58 | 11.61 | 15.55 | -2.30 | 16.92 |
| Wind speed (m/s) | 2015 | 4187929 | 0.58 | 2.65 | 2.88 | 0.22 | 1.42 | 1.89 | 8.34 | 53.65 |
|  | 2020 | 4788904 | 0.60 | 2.57 | 2.87 | 0.30 | 1.41 | 1.86 | 11.78 | 54.90 |
| Wind direction  (°)* | 2015 | 3530479 | 0.39 | 192.21 | 183.75 | 6.04 | 48.82 | 66.82 | 3.14 | 25.40 |
|  | 2020 | 3982816 | 0.40 | 190.69 | 185.10 | 5.47 | 48.58 | 66.60 | 2.87 | 25.48 |
| Daily Precipitation (mm) | 2015 | 233325 | 0.39 | 4.85 | 5.08 | 0.22 | 5.63 | 14.20 | 4.55 | 115.93 |
|  | 2020 | 305570 | 0.43 | 4.22 | 4.38 | 0.16 | 4.87 | 12.89 | 3.81 | 115.46 |
| Note: units for Mean Observation, Mean Simulation, MB, GE, and RMSE are shown below the name of each parameter in the first column.  *MB, GE, RMSE, NMB, and NME of wind direction are calculated with the consideration of the periodic nature of wind direction. For example, the difference between 1° and 359° is 2° instead of 358°. | | | | | | | | | | |

Supplementary Table 8 | Definition of eastern and western China for air quality model evaluation.

| Province | Region |
| --- | --- |
| Beijing | Eastern |
| Tianjin | Eastern |
| Hebei | Eastern |
| Shanxi | Eastern |
| Liaoning | Eastern |
| Jilin | Eastern |
| Heilongjiang | Eastern |
| Shanghai | Eastern |
| Jiangsu | Eastern |
| Zhejiang | Eastern |
| Anhui | Eastern |
| Fujian | Eastern |
| Jiangxi | Eastern |
| Shandong | Eastern |
| Henan | Eastern |
| Hubei | Eastern |
| Hunan | Eastern |
| Guangdong | Eastern |
| Guangxi | Eastern |
| Hainan | Eastern |
| Chongqing | Eastern |
| Sichuan | Eastern |
| Guizhou | Eastern |
| Yunan | Eastern |
| Shannxi | Eastern |
| Tibet | Western |
| Inner Mongolia | Western |
| Gansu | Western |
| Qinghai | Western |
| Ningxia | Western |
| Xinjiang | Western |
| Hong Kong | Nodata |
| Macao | Nodata |
| Taiwan | Nodata |

Supplementary Table 9 | Evaluation of simulated PM_2.5_ concentrations against ground observations on daily basis.

| Year | Region | R | Mean  Observation | Mean  Simulation | MB | RM  SE | NMB | NME | MFB | MFE |
| --- | --- | --- | --- | --- | --- | --- | --- | --- | --- | --- |
| 2015 | China | 0.6 | 51.8 | 53.4 | 1.6 | 41.5 | 3.1 | 52.0 | -8.5 | 56.5 |
|  | Eastern | 0.6 | 52.9 | 58.0 | 5.1 | 40.7 | 9.7 | 50.4 | 1.8 | 50.9 |
|  | Western | 0.3 | 43.7 | 18.1 | -25.6 | 47.2 | -58.5 | 67.4 | -88.7 | 100.5 |
| 2020 | China | 0.5 | 34.3 | 36.4 | 2.1 | 30.2 | 6.2 | 54.7 | -6.2 | 57.6 |
|  | Eastern | 0.6 | 34.6 | 39.4 | 4.7 | 29.0 | 13.6 | 53.5 | 4.2 | 52.6 |
|  | Western | 0.5 | 31.8 | 12.6 | -19.2 | 39.0 | -60.3 | 65.5 | -90.9 | 98.3 |

Note: units for Mean Observation, Mean Simulation, MB, and RMSE are μg/m^3^, and units for NMB, NME, MFB, and MFE are %. The definitions of the eastern and western parts of China are presented in Supplementary Table 8, which are consistent with Geng et al. [30].

Supplementary Table 10 | Evaluation of simulated MDA8 O_3_ concentrations against ground observations on daily basis in the warm season.

| Year | Region | R | Mean  Observation | Mean  Simulation | MB | RM  SE | NMB | NME | MFB | MFE |
| --- | --- | --- | --- | --- | --- | --- | --- | --- | --- | --- |
| 2015 | China | 0.5 | 102.7 | 106.8 | 4.1 | 43.7 | 4.0 | 32.0 | 5.1 | 34.7 |
|  | Eastern | 0.6 | 102.5 | 108.6 | 6.1 | 44.2 | 5.9 | 32.3 | 6.9 | 34.9 |
|  | Western | 0.4 | 104.3 | 92.8 | -11.5 | 38.8 | -11.0 | 29.5 | -8.5 | 33.0 |
| 2020 | China | 0.7 | 108.8 | 109.9 | 1.1 | 31.2 | 1.0 | 21.5 | 0.4 | 23.4 |
|  | Eastern | 0.7 | 108.8 | 110.2 | 1.3 | 30.8 | 1.2 | 21.4 | 0.9 | 23.6 |
|  | Western | 0.5 | 108.8 | 107.5 | -1.3 | 34.0 | -1.2 | 22.6 | -3.4 | 22.2 |

Note: units for Mean Observation, Mean Simulation, MB, and RMSE are μg/m^3^, and units for NMB, NME, MFB, and MFE are %. The definitions of the eastern and western parts of China are presented in Supplementary Table 8, which are consistent with Geng et al. [30].

Supplementary Table 11 | Evaluation of simulated PM_2.5_ concentrations against multisource fused PM_2.5_ on annual basis.

| Year | Region | R | Mean  Observation | Mean  Simulation | MB | RM  SE | NMB | NME | MFB | MFE |
| --- | --- | --- | --- | --- | --- | --- | --- | --- | --- | --- |
| 2015 | China (population-weighted) | 0.8 | 52.9 | 52.7 | -0.1 | 17.3 | -0.3 | 22.9 | -8.2 | 26.6 |
|  | China | 0.8 | 34.5 | 17.1 | -17.4 | 22.3 | -50.3 | 56.7 | -106.3 | 110.6 |
|  | Eastern | 0.9 | 39.6 | 33.5 | -6.1 | 13.6 | -15.5 | 27.9 | -35.6 | 45.0 |
|  | Western | 0.4 | 30.3 | 3.7 | -26.5 | 27.4 | -87.7 | 87.7 | -164.2 | 164.2 |
| 2020 | China (population-weighted) | 0.7 | 33.0 | 35.2 | 2.2 | 13.1 | 6.6 | 28.2 | -2.9 | 30.5 |
|  | China | 0.7 | 22.3 | 11.8 | -10.5 | 14.9 | -46.9 | 57.6 | -102.8 | 109.8 |
|  | Eastern | 0.8 | 26.1 | 22.9 | -3.1 | 10.2 | -12.0 | 32.4 | -32.6 | 48.1 |
|  | Western | 0.6 | 19.2 | 2.7 | -16.5 | 17.8 | -85.7 | 85.7 | -160.3 | 160.3 |

Note: units for Mean Observation, Mean Simulation, MB, and RMSE are μg/m^3^, and units for NMB, NME, MFB, and MFE are %. The definitions of the eastern and western parts of China are presented in Supplementary Table 8, which are consistent with Geng et al. [30].

Supplementary Table 12 | Evaluation of simulated MDA8 O_3_ against multisource fused MDA8 O_3_ on seasonal basis in the warm season.

| Year | Region | R | Mean  Observation | Mean  Simulation | MB | RM  SE | NMB | NME | MFB | MFE |
| --- | --- | --- | --- | --- | --- | --- | --- | --- | --- | --- |
| 2015 | China (population-weighted) | 0.7 | 97.6 | 110.6 | 13.0 | 20.7 | 13.3 | 17.3 | 11.8 | 16.0 |
|  | China | 0.7 | 88.1 | 91.5 | 3.4 | 15.7 | 3.9 | 13.8 | 2.5 | 13.1 |
|  | Eastern | 0.8 | 87.1 | 100.5 | 13.4 | 19.6 | 15.4 | 18.1 | 12.7 | 16.1 |
|  | Western | 0.6 | 89.0 | 84.2 | -4.8 | 11.6 | -5.4 | 10.4 | -5.9 | 10.7 |
| 2020 | China (population-weighted) | 0.9 | 111.9 | 111.8 | -0.1 | 10.9 | -0.1 | 7.8 | -0.2 | 8.1 |
|  | China | 0.8 | 102.2 | 100.7 | -1.5 | 11.9 | -1.5 | 9.5 | -2.0 | 9.8 |
|  | Eastern | 0.9 | 98.6 | 102.0 | 3.4 | 10.9 | 3.4 | 8.9 | 2.7 | 9.1 |
|  | Western | 0.5 | 105.2 | 99.7 | -5.5 | 12.8 | -5.2 | 9.9 | -5.8 | 10.3 |

Note: units for Mean Observation, Mean Simulation, MB, and RMSE are μg/m^3^, and units for NMB, NME, MFB, and MFE are %. The definitions of the eastern and western parts of China are presented in Supplementary Table 8, which are consistent with Geng et al. [30].

Supplementary Table 13 | Synergy Index calculated based on 2015 and 2020 baselines.

| Period | Year | Scenario | Synergy Index | | Percentage change |
| --- | --- | --- | --- | --- | --- |
|  |  |  | 2015 as baseline | 2020 as baseline |  |
| Period I | 2015 | All policy combined | 0.75 | 0.74 | -1.3% |
|  | 2015 | Strengthen Standard and Fuel Quality (SEQ) | 0.40 | 0.39 | -2.5% |
|  | 2015 | Phase Out Outdated Vehicle (POV) | 0.44 | 0.43 | -2.3% |
|  | 2015 | Improve Fuel Efficiency (IFE) | 0.35 | 0.34 | -2.9% |
|  | 2015 | Optimize Transportation Structure (OTS) | 0.25 | 0.25 | 0.0% |
|  | 2015 | Promote New Energy Vehicle (PNV) | 0.00 | 0.00 | 0.0% |
| Period II | 2020 | All policy combined | 0.61 | 0.60 | -1.6% |
|  | 2020 | Strengthen Standard and Fuel Quality (SEQ) | 0.25 | 0.25 | -4.0% |
|  | 2020 | Phase Out Outdated Vehicle (POV) | 0.27 | 0.27 | 0.0% |
|  | 2020 | Improve Fuel Efficiency (IFE) | 0.25 | 0.25 | 0.0% |
|  | 2020 | Optimize Transportation Structure (OTS) | 0.32 | 0.31 | -3.1% |
|  | 2020 | Promote New Energy Vehicle (PNV) | 0.14 | 0.13 | -7.1% |
| Counter-  factual | 2020 | Strengthen Vehicle Fleet Structure Transition (FleTran) | 0.52 | 0.51 | -1.9% |
|  | 2020 | Strengthen Transportation Structure Transition (TraTran) | 0.51 | 0.51 | 0.0% |
|  | 2020 | Combined Structure Transition (StrTran) | 0.79 | 0.78 | -1.3% |
| Note: Percentage change represents the relative difference between the Synergy Index with 2020 as baseline and that with 2015 as baseline. | | | | | |

**References**

1. National Bureau of Statistic of China. *China Statistics Yearbook (2010-2021)*. <http://www.stats.gov.cn/english/> (7 November 2024, date last accessed).

2. Zhang Q, Zheng Y, Tong D *et al.* Drivers of improved PM_2.5_ air quality in China from 2013 to 2017. *Proc Natl Acad Sci U S A*. 2019; **116**: 24463-24469. doi: 10.1073/pnas.1907956116

3. Geng G, Liu Y, Liu Y *et al.* Efficacy of China’s clean air actions to tackle PM_2.5_ pollution between 2013 and 2020. *Nat Geosci*. 2024; **17**: 987-994. doi: 10.1038/s41561-024-01540-z

4. Qi Z, Zheng Y, Feng Y *et al.* Co-drivers of air pollutant and CO_2_ emissions from on-road transportation in China 2010-2020. *Environ Sci Technol*. 2023; **57**: 20992-21004. doi: 10.1021/acs.est.3c08035

5. The International Council on Clean Transportation and DieselNet. *China light-duty fuel consumption*. <https://www.transportpolicy.net/standard/china-light-duty-fuel-consumption/> (10 February 2025, date last accessed).

6. The State Council of the People's Republic of China. *Transportation Development Statistics Bulletin (2011-2021)*. <http://www.gov.cn/xinwen/2022-05/25/content_5692174.htm> (10 November 2024, date last accessed).

7. Zhang S. *Technical Guidelines on Emission Inventory in China*. <http://dx.doi.org/10.13140/RG.2.1.3168.4005> (16 November 2024, date last accessed).

8. Wu X. Research on synergistic control strategies of air pollutants and CO_2_ emissions from road motor vehicles in China *Doctor of Philosophy Degree*. Tsinghua University, 2017.

9. Ministry of Ecology and Environment of the People's Republic of China. *China Mobile Source Environmental Management Annual Report (2011-2024)*. <http://english.mee.gov.cn/> (11 February 2025, date last accessed).

10. Zhang S, Wu Y, Liu H *et al.* Real-world fuel consumption and CO_2_ (carbon dioxide) emissions by driving conditions for light-duty passenger vehicles in China. *Energy*. 2014; **69**: 247-257. doi: 10.1016/j.energy.2014.02.103

11. Zhang S, Wu Y, Liu H *et al.* Historical evaluation of vehicle emission control in Guangzhou based on a multi-year emission inventory. *Atmos Environ*. 2013; **76**: 32-42. doi: 10.1016/j.atmosenv.2012.11.047

12. Wu Y, Wang R, Zhou Y *et al.* On-road vehicle emission control in Beijing: Past, present, and future. *Environ Sci Technol*. 2011; **45**: 147-153. doi: 10.1021/es1014289

13. Wu Y, Zhang S, Hao J *et al.* On-road vehicle emissions and their control in China: A review and outlook. *Sci Total Environ*. 2017; **574**: 332-349. doi: 10.1016/j.scitotenv.2016.09.040

14. Wu Y, Yang Z, Lin B *et al.* Energy consumption and CO_2_ emission impacts of vehicle electrification in three developed regions of China. *Energy Policy*. 2012; **48**: 537-550. doi: 10.1016/j.enpol.2012.05.060

15. Zhou J, Wang J, Jiang H *et al.* Cost-benefit analysis of yellow-label vehicles scrappage subsidy policy: A case study of Beijing-Tianjin-Hebei region of China. *J Clean Prod*. 2019; **232**: 94-103. doi: 10.1016/j.jclepro.2019.05.312

16. Yin C, Shao C, Wang X. Exploring the impact of built environment on car use: does living near urban rail transit matter? *Transp Lett*. 2020; **12**: 391-398. doi: 10.1080/19427867.2019.1611196

17. Jia T, Li Q, Shi W. Estimation and analysis of emissions from on-road vehicles in Mainland China for the period 2011-2015. *Atmos Environ*. 2018; **191**: 500-512. doi: 10.1016/j.atmosenv.2018.08.037

18. Zhang S, Wu Y, Wu X *et al.* Historic and future trends of vehicle emissions in Beijing, 1998-2020: A policy assessment for the most stringent vehicle emission control program in China. *Atmos Environ*. 2014; **89**: 216-229. doi: 10.1016/j.atmosenv.2013.12.002

19. Huo H, Zhang Q, He K *et al.* Vehicle-use intensity in China: Current status and future trend. *Energy Policy*. 2012; **43**: 6-16. doi: 10.1016/j.enpol.2011.09.019

20. Zheng B, Zhang Q, Zhang Y *et al.* Heterogeneous chemistry: a mechanism missing in current models to explain secondary inorganic aerosol formation during the January 2013 haze episode in North China. *Atmos Chem Phys*. 2015; **15**: 2031-2049. doi: 10.5194/acp-15-2031-2015

21. Li X, Zhang Q, Zhang Y *et al.* Source contributions of urban PM_2.5_ in the Beijing–Tianjin–Hebei region: Changes between 2006 and 2013 and relative impacts of emissions and meteorology. *Atmos Environ*. 2015; **123**: 229-239. doi: 10.1016/j.atmosenv.2015.10.048

22. Zheng Y, Cao W, Zhao H *et al.* Identifying key sources for air pollution and CO_2_ emission co-control in China. *Environ Sci Technol*. 2024. doi: 10.1021/acs.est.4c03299

23. Atmospheric Chemistry Observations & Modeling, National Center for Atmospheric Research, Research UCfA. CESM2.1 The Community Atmosphere Model with Chemistry (CAM-chem) Outputs as Boundary Conditions. NSF: National Center for Atmospheric Research. 2020. doi: 10.5065/CKR4-GP38

24. Tsinghua University. *The Multi-resolution Emission Inventory of China (MEIC)*. <http://meicmodel.org.cn/> (20 April 2024, date last accessed).

25. Li M, Zhang Q, Kurokawa JI *et al.* MIX: a mosaic Asian anthropogenic emission inventory under the international collaboration framework of the MICS-Asia and HTAP. *Atmos Chem Phys*. 2017; **17**: 935-963. doi: 10.5194/acp-17-935-2017

26. Liu Y, Geng G, Cheng J *et al.* Drivers of Increasing Ozone during the Two Phases of Clean Air Actions in China 2013-2020. *Environ Sci Technol*. 2023; **57**: 8954-8964. doi: 10.1021/acs.est.3c00054

27. Guenther AB, Jiang X, Heald CL *et al.* The Model of Emissions of Gases and Aerosols from Nature version 2.1 (MEGAN2.1): an extended and updated framework for modeling biogenic emissions. *Geosci Model Dev*. 2012; **5**: 1471-1492. doi: 10.5194/gmd-5-1471-2012

28. Emery CA, Tai E. *Enhanced Meteorological Modeling and Performance Evaluation for Two Texas Ozone Episodes*. <https://downloads.regulations.gov/EPA-R06-OAR-2008-0510-0015/attachment_11.pdf> (31 August 2024, date last accessed).

29. US Environmental Protection Agency. *Guidance on the Use of Models and Other Analyses for Demonstrating Attainment of Air Quality Goals for Ozone, PM_2.5_, and Regional Haze*. <https://www.epa.gov/sites/default/files/2020-10/documents/final-03-pm-rh-guidance.pdf> (25 April 2024, date last accessed).

30. Geng G, Zheng Y, Zhang Q *et al.* Drivers of PM_2.5_ air pollution deaths in China 2002-2017. *Nat Geosci*. 2021; **14**: 645-650. doi: 10.1038/s41561-021-00792-3

31. Liu S, Geng G, Xiao Q *et al.* Tracking daily concentrations of PM_2.5_ chemical composition in China since 2000. *Environ Sci Technol*. 2022; **56**: 16517-16527. doi: 10.1021/acs.est.2c06510

32. He Q, Gu Y, Zhang M. Spatiotemporal patterns of aerosol optical depth throughout China from 2003 to 2016. *Sci Total Environ*. 2019; **653**: 23-35. doi: 10.1016/j.scitotenv.2018.10.307

33. Geng G, Xiao Q, Liu S *et al.* Tracking air pollution in China: near real-time PM_2.5_ retrievals from multisource data fusion. *Environ Sci Technol*. 2021; **55**: 12106-12115. doi: 10.1021/acs.est.1c01863

34. Xiao Q, Geng G, Liu S *et al.* Spatiotemporal continuous estimates of daily 1 km PM_2.5_ from 2000 to present under the Tracking Air Pollution in China (TAP) framework. *Atmos Chem Phys*. 2022; **22**: 13229-13242. doi: 10.5194/acp-22-13229-2022

35. Xue T, Zheng Y, Geng G *et al.* Estimating Spatiotemporal Variation in Ambient Ozone Exposure during 2013-2017 Using a Data-Fusion Model. *Environ Sci Technol*. 2020; **54**: 14877-14888. doi: 10.1021/acs.est.0c03098

36. Xiao Q, Geng G, Xue T *et al.* Tracking PM_2.5_ and O_3_ Pollution and the Related Health Burden in China 2013-2020. *Environ Sci Technol*. 2022; **56**: 6922-6932. doi: 10.1021/acs.est.1c04548

37. Center for International Earth Science Information Network - CIESIN - Columbia University. Gridded Population of the World, Version 4 (GPWv4): Population Count Adjusted to Match 2015 Revision of UN WPP Country Totals, Revision 11. Palisades, New York: NASA Socioeconomic Data and Applications Center (SEDAC). 2018. doi: doi.org/10.7927/H4JW8BX5

38. Burnett RT, Pope CA, Ezzati M *et al.* An integrated risk function for estimating the Global Burden of Disease attributable to ambient fine particulate matter exposure. *Environ Health Perspect*. 2014; **122**: 397-403. doi: 10.1289/ehp.1307049

39. Tong D, Geng G, Zhang Q *et al.* Health co-benefits of climate change mitigation depend on strategic power plant retirements and pollution controls. *Nat Clim Chang*. 2021; **11**: 1077-1083. doi: 10.1038/s41558-021-01216-1

40. Cai W, Zhang C, Suen HP *et al.* The 2020 China report of the Lancet Countdown on health and climate change. *The Lancet Public Health*. 2021; **6**: e64-e81. doi: 10.1016/S2468-2667(20)30256-5

41. Burnett R, Chen H, Szyszkowicz M *et al.* Global estimates of mortality associated with long-term exposure to outdoor fine particulate matter. *Proc Natl Acad Sci U S A*. 2018; **115**: 9592-9597. doi: 10.1073/pnas.1803222115

42. Zheng Y, Xue T, Zhao H *et al.* Increasing life expectancy in China by achieving its 2025 air quality target. *Env Sci Ecotechnol*. 2022; **12**: 100203. doi: 10.1016/j.ese.2022.100203

43. Murray CJL, Aravkin AY, Zheng P *et al.* Global burden of 87 risk factors in 204 countries and territories, 1990-2019: a systematic analysis for the Global Burden of Disease Study 2019. *Lancet*. 2020; **396**: 1223-1249. doi: 10.1016/S0140-6736(20)30752-2

44. Brauer M, Roth GA, Aravkin AY *et al.* Global burden and strength of evidence for 88 risk factors in 204 countries and 811 subnational locations, 1990-2021: a systematic analysis for the Global Burden of Disease Study 2021. *Lancet*. 2024; **403**: 2162-2203. doi: 10.1016/S0140-6736(24)00933-4

45. GBD Maps Working Group. *Burden of disease attributable to coal-burning and other major sources of air pollution in China*. <https://www.healtheffects.org/publication/burden-disease-attributable-coal-burning-and-other-air-pollution-sources-china> (20 October 2024, date last accessed).

46. Zhang Q, Jiang X, Tong D *et al.* Transboundary health impacts of transported global air pollution and international trade. *Nature*. 2017; **543**: 705-709. doi: 10.1038/nature21712

47. Archer-Nicholls S, Carter E, Kumar R *et al.* The regional impacts of cooking and heating emissions on ambient air quality and disease burden in China. *Environ Sci Technol*. 2016; **50**: 9416-9423. doi: 10.1021/acs.est.6b02533

48. Huang ZN, Jia HH, Shi XH *et al.* Revealing the impact of China's clean air policies on synergetic control of CO_2_ and air pollutant emissions: Evidence from Chinese cities. *J Environ Manage*. 2023; **344**. doi: 10.1016/j.jenvman.2023.118373

49. Chen XL, Meng QG, Wang KK *et al.* Spatial patterns and evolution trend of coupling coordination of pollution reduction and carbon reduction along the Yellow River Basin, China. *Ecol Indic*. 2023; **154**. doi: 10.1016/j.ecolind.2023.110797

50. Wang YW, Ni JM, Xu KW *et al.* Intricate synergistic effects between air pollution and carbon emission: An emerging evidence from China. *Environ Pollut*. 2024; **349**. doi: 10.1016/j.envpol.2024.123851

51. The State Council of the People's Republic of China. *The 14th Five-Year Plan of the People’s Republic of China*. <https://www.gov.cn/xinwen/2021-03/13/content_5592681.htm> (10 May 2024, date last accessed).

52. The State Council of the People's Republic of China. *Work Plan for Promoting the Development of Multimodal Transportation and Optimizing and Adjusting the Transportation Structure (2021-2025)*. <https://www.mee.gov.cn/zcwj/gwywj/202201/t20220110_966519.shtml> (28 August 2024, date last accessed).

53. The State Council of the People's Republic of China. *Action Plan for Carbon Dioxide Peaking Before 2030*. <https://www.gov.cn/zhengce/content/2021-10/26/content_5644984.htm> (28 August 2024, date last accessed).

54. Huang Z, Ji L, Yin J *et al.* Peak pathway of China's road transpportation carbon emissions. *Research of Environmental Sciences*. 2022; **35**: 385-393. doi: 10.13198/j.issn.1001-6929.2021.11.06

55. Zheng B, Tong D, Li M *et al.* Trends in China's anthropogenic emissions since 2010 as the consequence of clean air actions. *Atmos Chem Phys*. 2018; **18**: 14095-14111. doi: 10.5194/acp-18-14095-2018

56. Crippa M, Guizzardi D, Muntean M *et al.* *EDGAR v6.1 global air pollutant emissions*. <https://publications.jrc.ec.europa.eu/repository/handle/JRC129555> (30 November 2024, date last accessed).

57. O’Rourke P, Smith S, Mott A *et al.* CEDS V_2021_02_05 Release Emission Data. 2021. doi: zenodo.org/record/192

58. Cozzi L, Gould T, Bouckart S *et al.* *World energy outlook 2020*. <https://www.iea.org/reports/world-energy-outlook-2020> (18 November 2024, date last accessed).

59. Wen Y, Liu M, Zhang S *et al.* Updating on-road vehicle emissions for China: spatial patterns, temporal trends, and mitigation drivers. *Environ Sci Technol*. 2023; **57**: 14299-14309. doi: 10.1021/acs.est.3c04909

60. Tanaka K, Cavalett O, Collins WJ *et al.* Asserting the climate benefits of the coal-to-gas shift across temporal and spatial scales. *Nat Clim Chang*. 2019; **9**: 389-396. doi: 10.1038/s41558-019-0457-1

61. Fry MM, Naik V, West JJ *et al.* The influence of ozone precursor emissions from four world regions on tropospheric composition and radiative climate forcing. *J Geophys Res-Atmos*. 2012; **117**. doi: 10.1029/2011JD017134

62. Collins WJ, Fry MM, Yu H *et al.* Global and regional temperature-change potentials for near-term climate forcers. *Atmos Chem Phys*. 2013; **13**: 2471-2485. doi: 10.5194/acp-13-2471-2013

63. Yu H, Chin M, West JJ *et al.* A multimodel assessment of the influence of regional anthropogenic emission reductions on aerosol direct radiative forcing and the role of intercontinental transport. *J Geophys Res-Atmos*. 2013; **118**: 700-720. doi: 10.1029/2012JD018148

64. Gasser T, Peters GP, Fuglestvedt JS *et al.* Accounting for the climate–carbon feedback in emission metrics. *Earth Syst Sci Data*. 2017; **8**: 235-253. doi: 10.5194/esd-8-235-2017

65. Tibrewal K, Venkataraman C. Climate co-benefits of air quality and clean energy policy in India. *Nat Sustain*. 2021; **4**: 305-313. doi: 10.1038/s41893-020-00666-3

66. Wang H, He X, Liang X *et al.* Health benefits of on-road transportation pollution control programs in China. *Proc Natl Acad Sci U S A*. 2020; **117**: 25370-25377. doi: 10.1073/pnas.1921271117

67. Liu M, Wen Y, Wu X *et al.* Province-level decarbonization potentials for China’s road transportation sector. *Environ Sci Technol*. 2024. doi: 10.1021/acs.est.4c06755

68. Liu M, Lei Y, Wang X *et al.* Source contributions to PM_2.5_-related mortality and costs: evidence for emission allocation and compensation strategies in China. *Environ Sci Technol*. 2023; **57**: 4720-4731. doi: 10.1021/acs.est.2c08306

69. Giani P, Anav A, De Marco A *et al.* Exploring sources of uncertainty in premature mortality estimates from fine particulate matter: the case of China. *Environ Res Lett*. 2020; **15**. doi: 10.1088/1748-9326/ab7f0f

70. The International Council on Clean Transportation and DieselNet. *China heavy-duty fuel consumption*. <https://www.transportpolicy.net/standard/china-heavy-duty-fuel-consumption/> (10 February 2025, date last accessed).

71. International Energy Agency. *Global EV Outlook 2021*. <https://www.iea.org/reports/global-ev-outlook-2021> (20 February 2025, date last accessed).
